# m^6^A-Driver: Identifying Context-Specific mRNA m^6^A Methylation-Driven Gene Interaction Networks

**DOI:** 10.1371/journal.pcbi.1005287

**Published:** 2016-12-27

**Authors:** Song-Yao Zhang, Shao-Wu Zhang, Lian Liu, Jia Meng, Yufei Huang

**Affiliations:** 1 School of Automation, Northwestern Polytechnical University, Xi'an, Shaanxi, China; 2 Department of Biological Sciences, HRINU, SUERI, Xi’an Jiaotong-Liverpool University, Suzhou, Jiangsu, China; 3 Department of Electrical and Computer Engineering, University of Texas at San Antonio, San Antonio, Texas, United States of America; Ottawa University, CANADA

## Abstract

As the most prevalent mammalian mRNA epigenetic modification, N6-methyladenosine (m^6^A) has been shown to possess important post-transcriptional regulatory functions. However, the regulatory mechanisms and functional circuits of m^6^A are still largely elusive. To help unveil the regulatory circuitry mediated by mRNA m^6^A methylation, we develop here m^6^A-Driver, an algorithm for predicting m^6^A-driven genes and associated networks, whose functional interactions are likely to be actively modulated by m^6^A methylation under a specific condition. Specifically, m^6^A-Driver integrates the PPI network and the predicted differential m^6^A methylation sites from methylated RNA immunoprecipitation sequencing (MeRIP-Seq) data using a Random Walk with Restart (RWR) algorithm and then builds a consensus m^6^A-driven network of m^6^A-driven genes. To evaluate the performance, we applied m^6^A-Driver to build the context-specific m^6^A-driven networks for 4 known m^6^A (de)methylases, i.e., FTO, METTL3, METTL14 and WTAP. Our results suggest that m^6^A-Driver can robustly and efficiently identify m^6^A-driven genes that are functionally more enriched and associated with higher degree of differential expression than differential m^6^A methylated genes. Pathway analysis of the constructed context-specific m^6^A-driven gene networks further revealed the regulatory circuitry underlying the dynamic interplays between the methyltransferases and demethylase at the epitranscriptomic layer of gene regulation.

## Author Summary

Powered by methylated RNA immunoprecipitation sequencing (MeRIP-Seq) technology, recent studies have revealed a new mode of post transcriptional regulation mediated by mRNA N6-methyladenosine (m^6^A). Currently, the analysis of m^6^A focuses mostly on prediction of m^6^A sites as well as differential m^6^A methylation, and systematic approach for predicting m^6^A functions is yet to emerge. We develop here m^6^A-Driver, the first network-based approach, to identify m^6^A-driven genes and their associated networks, whose functional interactions are likely to be actively modulated by m^6^A methylation under a specific condition. Our test results showed that m^6^A-Driver can robustly and efficiently identify m^6^A-driven genes that are functionally more enriched and associated with higher degree of differential expression than differential m^6^A methylated genes. m^6^A-Driver is an effective and reliable approach to identify functionally relevant m^6^A-driven genes and networks from MeRIP-Seq data.

## Introduction

Methylation, as a significant epigenetic modification of nucleic acids, regulates gene expression, influences grows and development of plants and animals, and is closely related to the occurrence and development of disease. The epigenetic regulatory mechanisms and physiological functions of DNA methylation have been well established through intensive studies in simple model organisms to human in the past decade [1–3]. However, RNA methylation, even though prevalent in many organisms, has long been considered to have little functional relevance. The discovery of obesity-associated FTO as a demethylase [4] of mRNA N6-methyladenosine (m^6^A) revealed that mRNA m^6^A methylation can be reversed and is thus a highly dynamic phenomenon. This discovery sparked the surged interests in study the prevalence of m^6^A in different cells and the functions of m^6^A. Subsequently, using methylated RNA immunoprecipitation sequencing (MeRIP-seq) technique [5–7], transcriptome-wide distribution of m^6^A in mammalian cells was profiled [6, 7], revealing for the first time a widespread occurrence of m^6^A in >25% transcripts. m^6^A was also shown to be enriched around the stop codon of RNA transcripts and conserved between people and mouse [6, 7], implicating a potential role played by m^6^A in post-transcriptional regulation [6, 8, 9]. Since then, m^6^A has been shown to have a number of important biological functions, including promoting RNA degradation [10], regulating RNA stability by modulating binding of RNA binding proteins [6, 11, 12], and controlling translation efficiency [13–17]. Meanwhile, the identification of m^6^A methyltransferases and demethylases [4, 18–20] further revealed the regulators of epitranscriptome. We now know that the m^6^A methyltransferase complex consists of METTL3, METTL14, and WTAP and functions as m^6^A "writers" in eukaryotes [9, 18, 21]. In contrast, FTO and ALKBH5 are identified to be de-methyltransferase, or m^6^A "erasers" [4, 9, 22], indicating that mRNA m^6^A methylation is a dynamic process [4] and directly regulated by a number of methylases and demethylases [23]. Knockdown studies of these (de)methylases further revealed their involvement in many significant physiological processes including obesity [24–26], synaptic signaling [27], cancer [28, 29], sperm development [22], stem cell differentiation [30], circadian periods [31], yeast meiosis [32, 33], and stem cell pluripotency [34–36]. Although these studies together greatly improve our understanding of the reversible mRNA m^6^A methylation, the regulatory mechanisms and functional circuitry of m^6^A are still largely elusive.

Currently, MeRIP-Seq is the most widely adopted high throughput approach for measuring transcriptome-wide m^6^A methylation [6, 7, 37]. To obtain a transcriptome-wide m^6^A profile, MeRIP-Seq produces two sets of samples, i.e., IP and input samples. While IP samples include sequencing reads from m^6^A methylated RNA fragments pulled down with anti-m^6^A antibody, input samples measure the basal abundance of all transcripts, which are used as background for assessing the enrichment of methylated fragment. Detecting m^6^A methylation site or "peak detection" from MeRIP-Seq data can be achieved by comparing the enrichment of reads in the IP samples vs. those in the input samples. Several algorithms including exomePeak have been developed for m^6^A peak detection [38–41]. After the methylation sites are identified, differential m^6^A methylation (DmM) analysis can be also performed in a case-control study to further identify the dynamic m^6^A sites whose methylation status is significantly different under two experimental conditions. Algorithms such as exomePeak [42] and MeTDiff [43] have also been developed for this purpose. While peak detection and DmM analysis are essential steps for m^6^A bioinformatics analysis, they do not yet provide direct information about the functional relevance of m^6^A.

We focus in this paper on predicting m^6^A-driven genes (mDrGenes) and the m^6^A-driven gene interaction network (mDrNet). Specifically, we refer mDrGenes as genes whose mRNAs harbor DmM sites or differential m^6^A methylation genes (DmMGs), and thus may be under dynamic epitranscriptomic regulation and be functionally significant to the biological context of interest. Conceivably, when data is available, mDrGenes can be conveniently identified by first predicting the DmMGs and then assessing their functional significance by using functional networks such as Protein-Protein Interactions (PPI) network or biological pathways. However, identifying functionally significant DmMGs when there are replicates can be nontrivial. The challenge arises as a result of technical and biological bias, where significant DmMGs identified in some replicates might not be significant in other replicates. Existing algorithms for DmM analysis such as exomePeak and MeTDiff all devise different methods ranging from taking consensus DmM sites [42] to statistically modeling of replicate samples [43] to mitigate this bias. While they can help detect robust DmM sites, these DmM sites might not be functionally significant DmMGs. As our goal emphasizes on detecting functional significance, an approach that can address this bias in assessing functional significance is more desirable and likely to better identify the m^6^A-driven genes and network.

To address the aforementioned issue, we propose in this paper m^6^A-Driver, an algorithm that predicts mDrGenes by evaluating the consistency of RNA differential methylation from a functional network perspective. Specifically, rather than predicting DmMGs directly, m^6^A-Driver first performs DmM analysis on every possible replicate set (RS) independently, where each RS includes two IP-input pairs, one from the treated/case condition and the other from the untreated/control condition. Then, a DmM functional network is constructed for each RS by searching the significant interactions with DmMGs in PPI network using a Random Walk with Restart (RWR) algorithm. We adopt PPI network here to model functional interactions of m^6^A mediating genes because m^6^A has been shown to regulate the process of translation [13–17], in addition to its influence on gene expression. Finally, a consensus m^6^A-driven gene network is built by taking all the significant reoccurring interactions. By assessing the consensus among RS networks as opposed to RS DmMGs, m^6^A-Driver effectively addresses the sample bias that impacts functional prediction.

m^6^A-Driver was applied to four case-control studies that investigate the functions of the component of methyltransferase complex (METTL3, METTL14, and WTAP) and demethylase (FTO). In the end, m^6^A-driven gene networks were constructed for each (de)methylase together with an integrated network for the complete m^6^A methyltransferase complex. We showed that the predicted m^6^A-driven genes have higher degree of differential expression and more explicit functional relevance than DmMGs identified directly by previous approaches. These results demonstrate the effectiveness of m^6^A-Driver in prioritizing functional significant m^6^A-driven genes from m^6^A sequencing data.

## Results

### An overview of the m^6^A-Driver algorithm

**Fig 1:**
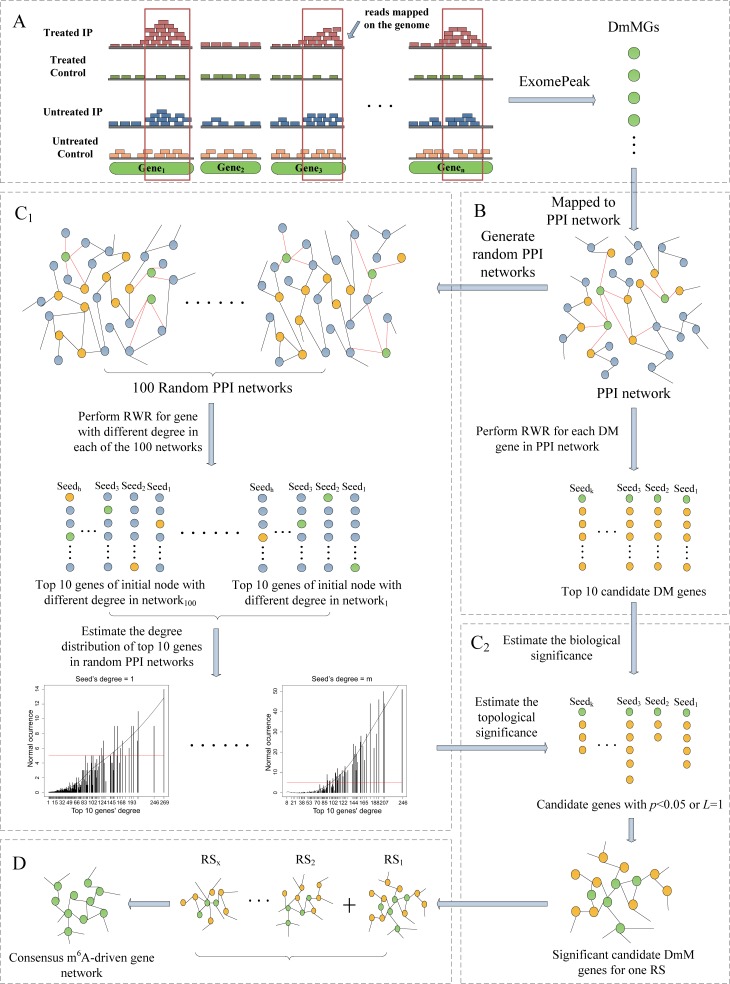
Flowchart of m^6^A-Driver. The green points denote DmMGs; the yellow points denote candidate DmMGs (DmM interacting genes) and the gray points represents the rest other genes. (A) DmMGs are detected by exomePeak in each RS. (B) Each DmMG is mapped to the PPI network and then serves as the starting node to initiate the RWR and its top accessible nodes are held as candidate genes. (C_1_) For a DmMG with specific degree, we retrieve the normalized degree distribution of its top accessible nodes in each of the 100 random networks, which are generated with the same degree distribution with the original network. The normal occurrence frequency of certain degree indicates the probability that a node with this degree to be selected by chance. (C_2_) The candidate genes are assessed by their topological and biological significance. Candidate genes that are not observed by random chance, i.e., *p*<0.05, or are biological significant, i.e., *L* = 1, are retained as significant candidate genes. (D) A consensus m^6^A-driven gene network is constructed by interactions recurring across all RSs.

The algorithm of m^6^A-Driver consists of four steps, depicted in Fig 1, with the first three steps implemented in each RS and step 4 performed to combine the results from all RSs. In step 1, exomePeak [42] is applied to detect DmMGs in each RS. In step 2, for each RS, the Random Walk with Restart (RWR) is performed using every DmMG as the seed node separately to search for their closely interacting genes in the PPI network. In step 3, the topological and biological significance of these DmM interacting genes are assessed and the genes that are determined to be insignificant are filtered out. The topological significance is estimated by their occurrence as top nodes prioritized by the same RWR algorithm in 100 random networks generated with the same topological structures. Meanwhile, the biological significance is evaluated by the length of their shortest path to the initial node (the seed DmMG). RS-specific DmM interacting networks, each consisting of significant interacting genes, are constructed at the end of step 3. Finally, in step 4, an mDrNet is constructed by assessing the interaction recurrence across all RSs. The genes that make up the nodes of the mDrNet are predicted mDrGenes determined by exomePeak. In this way, we extract a set of mDrGenes, or functionally relevant genes driven by m^6^A and a network that depicts the functional relationship of mDrGenes.

### An overview of the data

We applied m^6^A-Driver on 4 MeRIP-seq datasets, i.e., FTO knockdown dataset (KD-FTO) [23], METTL3 knockdown dataset (KD-METTL3), METTL14 knockdown dataset (KD-METTL4), and WTAP knockdown dataset (KD-WTAP) [44]. KD-FTO dataset is obtained from [27] that profiles m^6^A in FTO gene knockdown mice and their wild-type littermate. There are 12 samples (3 IP replicates paired with 3 input replicates for FTO knockdown mice and 3 IP replicates paired with 3 input replicates under wild-type littermate). It was divided into 9 sets of biological replicates and each biological replicate set (RS) contains two IP samples respectively from a FTO knockdown mouse and a wild-type (WT) littermate and two corresponding input samples from the two mice.

KD-METTL3, KD-METTL14 and KD-WTAP datasets are from a recent study, which shows that m^6^A regulates mRNA stability [44]. Each dataset contains 8 samples, 2 IP replicates paired with 2 input replicates from the knockdown HeLa cells and 2 IP replicates paired with 2 input replicates from untreated HeLa cells. Similar to KD-FTO, samples in each of the three datasets are then divided into 4 RSs, each of which contains two IP samples from the knockdown HeLa cells and untreated HeLa cells respectively and two corresponding input samples.

We first predicted the DmM sites in each dataset using exomePeak. As the technical limitation of MeRIP-Seq can lead to high sample bias, making the prediction results less reliable, we then set out to check the quality of the prediction results. First of all, the specificity of the predictions by exomePeak and MeTDiff on these datasets has been evaluated in a previously published paper [43], which shows that the false positive rates for all these datasets can be controlled and there are high specific DmM sites predicted in all these datasets. Next, we further examined the predictions of the three m^6^A methylase knockdown datasets, where we created a set of pseudo control and pseudo knockdown sequencing samples by scrambling the samples of a dataset so that the pseudo control and knockdown samples are both made up by a real control replicate and a real knockdown replicate. We then performed exomePeak on both the real dataset and the pseudo dataset and examined the prediction specificity at different thresholds by comparing the ratio of predicted DmM sites (or reported true positive rate, RTPR) in the real dataset and those in the pseudo dataset (or false positive rate, FPR) using a ROC-like curve. As is shown in S1 Fig, the percentage of DmM sites in the real datasets is much higher than that in the pseudo dataset at different thresholds in all the 3 datasets. Taken together, these results demonstrate that the false positive rates in these datasets can be controlled and the exomePeak prediction results are of good specificity for subsequent analysis.

The reference network, PPI network, is built from the most recent version of PPI data from BioGRID (release 3.4.128, compiled on August 25th, 2015) [45]. Based on the binary interactions, we removed the isolated proteins and self-interaction proteins to establish a PPI network with a total of 16,062 proteins and 152,676 interactions.

### m^6^A-Driver filters candidate DmMGs in a more robust and efficient way

Jia et al. have proposed the VarWalker algorithm [46] to combine PPI network and mutation data identified by next-generation sequencing (NGS) to build consensus networks for identifying cancer driver genes. While VarWalker was proposed for predicting driver mutations, it provides a general framework for prioritizing target genes from high-throughput sequencing data assisted by PPI network. VarWalker evaluates candidate target genes (i.e., mutation gene in cancer or DmMG in this work) by assessing their topological significance using random networks which hold the same degree distribution with the PPI network. However, utilizing only the topological characteristics may remove functionally significant candidate target genes. Also, the filtering result is not steady because it will remove different candidate genes for the same target gene when using different random networks. That is, VarWalker is not robust enough. We propose in this paper an improved strategy to evaluate both the topological and functional significance of candidate DmMGs in a more robust and efficient way, and the approach is detailed in the Materials and Methods section.

To compare the robustness and efficiency of m^6^A-Driver and VarWalker in filtering candidate genes, we applied the two methods on 100 genes randomly selected from the DmMGs in KD-METTL3 dataset to filter their candidate genes using two different sets of random networks. Each set contains 100 networks which hold the same topological property of PPI network. A more robust algorithm should remove a consistent set of genes in two random network sets. As is shown in Table 1, m^6^A-Driver only removed 1 different candidate gene when using different random network sets, whereas VarWalker removed 40 different candidate genes. This result demonstrates that m^6^A-Driver is more robust in filtering candidate genes. It is not surprising to also notice that some of the removed genes by VarWalker have significant biological functional connections with the seed (the DmMG) in the PPI network. Moreover, m^6^A can filter candidate genes in a more efficient way. VarWalker needs to perform RWR for each DmMG in each of the 100 random networks to compute the reoccurrence frequency of the candidate genes for calculating the p-value. In contract, m^6^A-Driver only needs the degree of a candidate gene and the degree of the seed gene to calculate its empirical p-value.

**Table 1:** Comparison of m^6^A-Driver and VarWalker.

Algorithm	#RG by RNS1	#RG by RNS2	#DRG for each seed	Running time
m^6^A-Driver	97	96	1	0.10s
VarWalker	241	229	40	37.02min

**#**RG represents the number of gene removed, RNS represents the random network set and **#**DRG represents the number of differential gene removed between RNS1 and RNS2. While VarWalker filters 40 different candidate genes using different RNSs, m^6^A-Driver removes only 1 candidate genes. Also, it takes 37.02 minutes for VarWalker to filter the candidate genes for 100 DmMGs but it takes only 0.1 second for m^6^A-Driver. This result demonstrates that m^6^A-Driver is more robust and efficient in filtering candidate DmMGs.

### The predicted mDrGenes closely interact with each other in the m^6^A-driven gene network

To validate m^6^A-Driver, we applied it to the four different case-control MeRIP-seq datasets: KD-FTO, KD-METTL3, KD-METTL14 and KD-WTAP. KD-FTO includes 9 RSs, based on which an FTO knockdown mDrNet (S2 Fig) was built. The network consists of 1,832 mDrGenes and 21,506 edges, with the maximal connected sub-graph containing 1,787 mDrGenes, implying that there exist dense interactions among mDrGenes. KD-METTL3, KD-METTL14 and KD-WTAP all include 4 RSs, based on which the corresponding context-specific mDrNets (S3–S5 Figs) were constructed by m^6^A-Driver. KD-METTL3 mDrNet contains 1,352 mDrGenes and 8,235 edges, with the maximal connected sub-graph including 1,339 mDrGenes; KD-METTL14 mDrNet consists of 1,251 mDrGenes and 8,452 edges, with itself being the maximal connected sub-graph; KD-WTAP mDrNet has 375 mDrGenes and 1,980 edges, which is also its maximal connected sub-graph. Similar to KD-FTO network, most mDrGenes in each of the 3 networks are interacting with each other very closely, implying again that the predicted mDrGenes have highly relevant functions.

### Characteristics of the predicted mDrGenes

**Fig 2:**
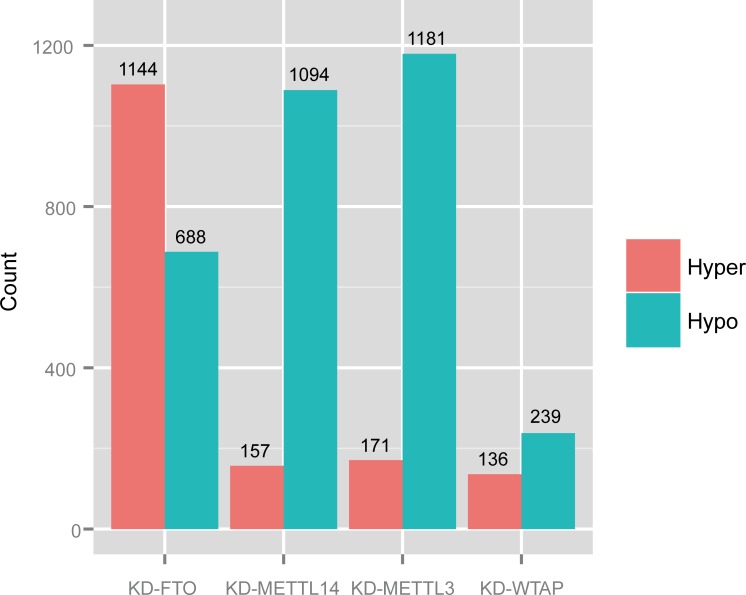
Counts of hyper/hypo mDrGene. As is expected, there are more hyper mDrGenes in KD-FTO and more hypo mDrGenes in KD-METTL3, KD-METTL14 and KD-WTAP.

**Fig 3:**
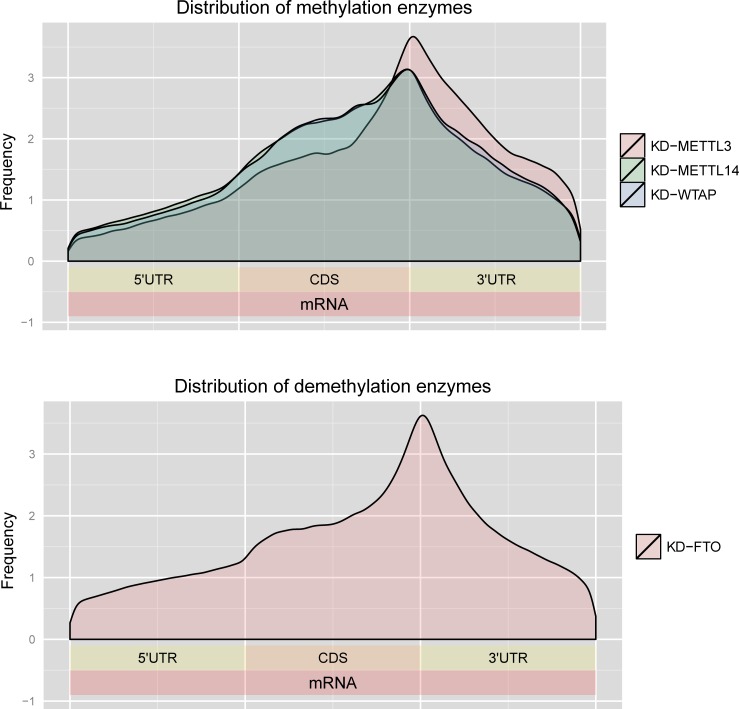
Distribution of DmM sites in mDrGenes. All the sites are consistently enriched in the 3'UTR and CDS in the 4 datasets. The plots was generated using the Guitar R/Bioconductor package [47].

We next examined the characteristics of the predicted mDrGenes. We first investigated the differential methylation of the mDrGenes. An mDrGenes is defined as a hyper mDrGenes if its most differentially methylated site is hyper-methylated, but otherwise defined as a hypo mDrGenes if its most differentially methylated site is hypo-methylated. We counted the number of hyper and hypo mDrGenes (Fig 2**)**. As expected, mDrGenes in KD-FTO are mostly hyper-methylated, whereas those in three other methylase knockdown datasets are more hypo-methylated. This result is consistent with the fact that FTO is an m^6^A demethylase, but METTL3, METTL14, and WTP are elements of m^6^A methyltransferase complex. We also calculated the average number of DmM sites for per gene and found that on average, an mDrGene harbors more than one DmM sites (Table 2). It is interesting that mDrGenes in KD-FTO harbor more DmM sites than the other 3 datasets and KD-METTL3 mDrGenes harbor the least number of DmM sites on average. We then investigated the DmM site distribution using the Guitar R/Bioconductor package [47] in an mDrGene transcript (Fig 3). Overall, the distributions for the 4 datasets are very similar, where DmM sites are mostly enriched around the stop codon and are distributed more in 3'UTR and CDS, which is consistent with the reported results in the literature [6, 7].

**Table 2:** Numbers of DmM sites and genes, and the average number of site per gene.

Dataset	# Sites	# Genes	Avg. sites/gene
KD-FTO	10204	1832	5.57
KD-METTL3	2684	1352	1.99
KD-METTL14	3450	1251	2.76
KD-WTAP	804	375	2.14

**Fig 4:**
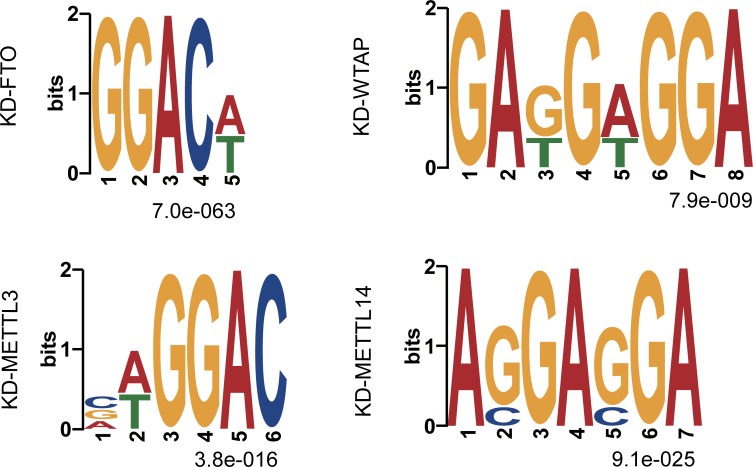
Sequence motifs of the DmM sites in mDrGenes. The motifs were identified using MEME-ChIP webserver. The shown motifs are the most enriched motifs in each dataset.

Furthermore, we obtained the sequence motifs of DmM sites in mDrGenes for each of the four datasets using MEME-ChIP webserver [48] (Fig 4). The reported RRACH m^6^A motifs [6, 7] was top ranked in KD-FTO and KD-METTL3, whereas the most enriched motifs in KD-METTL14 and KD-WTAP are similar to the binding motifs of SRSF1 and SRSF9. Interestingly, SRSF1 and SRSF9 are components of the SRSF protein that is involved in splice site selection in alternative splicing [49].

### mDrGenes are more functionally significant

**Fig 5:**
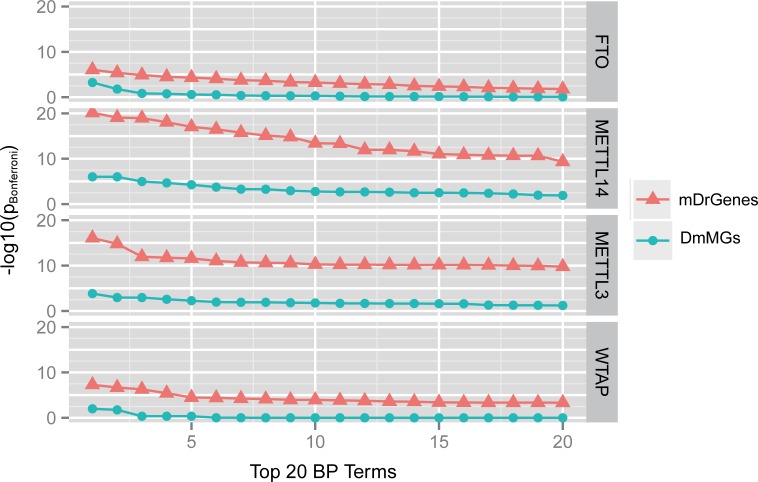
Comparison of functional enrichment between mDrGenes predicted by m^6^A-Driver and DmMGs by exomePeak. The figure shows the top 20 mostly enriched biological processes in mDrGenes and DmMGs, respectively. We can see that mDrGenes identified by m^6^A-Driver clearly have higher functional enrichment for all four datasets.

**Fig 6:**
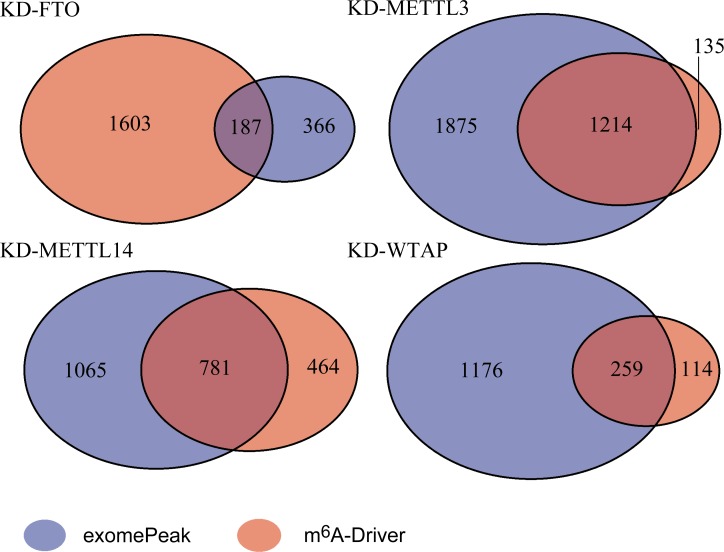
Number of mDrGenes predicted by m^6^A-Driver and DmMGs identified by exomePeak in four datasets. We can see that exomePeak predicts more genes in the three methylase knockdown datasets but m^6^A-Driver can find genes missed by exomePeak due to biological variance.

We asked if mDrGenes are more functional relevant. To test this, we examined the functionally significance between mDrGenes and DmMGs predicted by exomePeak. We performed GO [50] enrichment analysis using DAVID (Database of Annotation, Visualization and Integrated Discovery) [51] and then compared the enrichment degrees of the top enriched biological processes (BP, Fig 5). Since a larger testing gene set tends to lead to a smaller enriched p-value when performing DAVID, to make the comparison fair, we balanced the scale of mDrGenes and DmMGs before enrichment analysis. For KD-FTO dataset, the scale of mDrGenes is larger (Fig 6), so we randomly removed some mDrGenes to make the scales the same and then performed the enrichment analyses for 10 times to calculate an average p_Bonferroni_ for each enriched term. After also performing enrichment analysis on DmMGs, we compared the p_Bonferroni_ of top 20 enriched terms for DmMGs and scaled mDrGenes. Since, DmMGs have a larger scale for KD-METTL3, KD-METTL14 and KD-WTAP datasets, to balance the scale, we selected DmMGs that harbor top differently methylated DmM sites. Then, we performed enrichment analyses and compared the p_Bonferroni_ of top 20 enriched terms for the mDrGenes and scaled DmMGs. The result shows that mDrGenes are more significantly enriched than DmMGs in all the top enriched biological processes, demonstrating that mDrGenes are more functional relevant than DmMGs.

**Fig 7:**
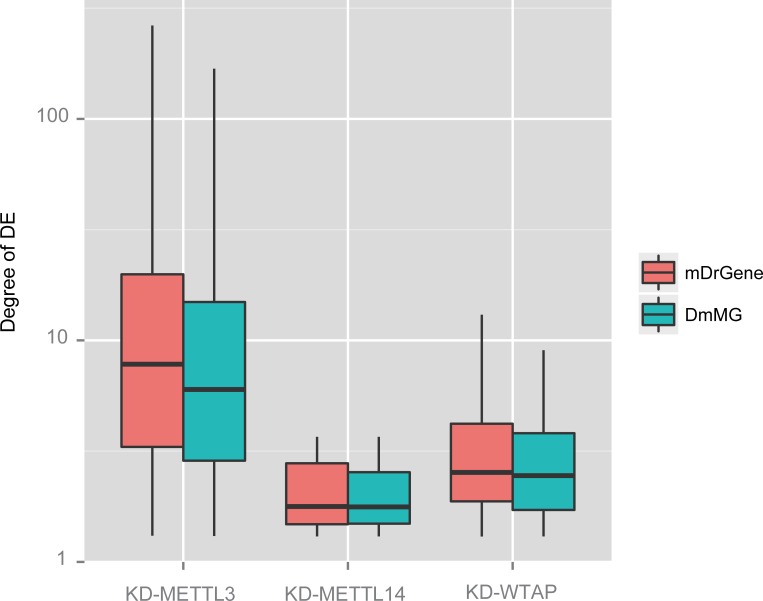
Degree of differential expression between mDrGenes and DmMGs. The degree is denoted by the negative log10 (FDR) calculated by DESeq2. We can see that the DE degree of mDrGenes is higher than that of DmMGs.

To further investigate the biological significance of mDrGenes, we evaluated the differential expression (DE) of mDrGenes and DmMGs. Conceivably, a gene set is likely to be more functionally important if it has more differentially expressed genes (DEGs) and/or its DEGs are more differentially expressed. To this end, we applied DESeq2 [52] to the input replicates of treated and untreated samples and determined a gene to be DEG if the adjusted *p*-value is less than 0.05. We first examined the percentage of DEGs in mDrGenes and DmMGs in the four datasets. We found that there are very few DEGs in mDrGenes and DmMGs for both KD-FTO and KD-METTL14 dataset, and thus the percentages of DEGs in mDrGenes and DmMGs are very low for these two datasets. Not surprisingly, no significant differences between the percentages of DEGs in mDrGenes and DmMGs can be observed (Fisher’s test, see Table 3 for details). In contrast, much more mDrGenes and DmMGs are differential expressed in KD-METTL3 and KD-WTAP, and the percentages in mDrGenes are significantly higher than those in DmMGs (Fisher’s test, see Table 3 for details). We next compared the degree of DE, which is represented by the negative log10 (FDR) calculated by DESeq2 (Fig 7). The result shows that the DE degrees of mDrGenes are also higher than those of DmMGs. The only exception is the FTO KD experiment, in which there are nearly no differential expression genes. Taken together, we can conclude that mDrGenes are likely to include more DEGs than DmMGs and their degree of DEs are also likely to be higher.

**Table 3:** Count of differently expressed genes (DEGs) among mDrGenes and DmMGs.

**Dataset**	**#DEG**	**DmMG**			**mDrGene**			**Fisher's test *p*-value**		
		#DE	#all	%DEG	#DE	#all	%DEG	2-tailed	greater	less
**KD-FTO**	12	1	553	0.18%	0	1832	0	0.23	1	0.23
**KD-M3**	6197	1850	3089	59.89%	901	1352	**66.64%**	1.98E-5	**1.06E-5**	1
**KD-M14**	127	30	1846	1.63%	22	1251	1.76%	0.78	0.44	0.67
**KD-WTAP**	1575	271	1435	18.89%	103	375	**27.47%**	4.30E-4	**2.32E-4**	1.00

M3 is short for METTL3 and M14 is short for METTL14. “2-tailed” represents the2-tailed Fisher's test, “greater” (or “less”) represents a one-tailed Fisher's test, where the null hypothesis is that the percentage of DEG in mDrGene is greater (or less) than that of DmMGs.

### mDrGenes participate in several important biological processes

**Fig 8:**
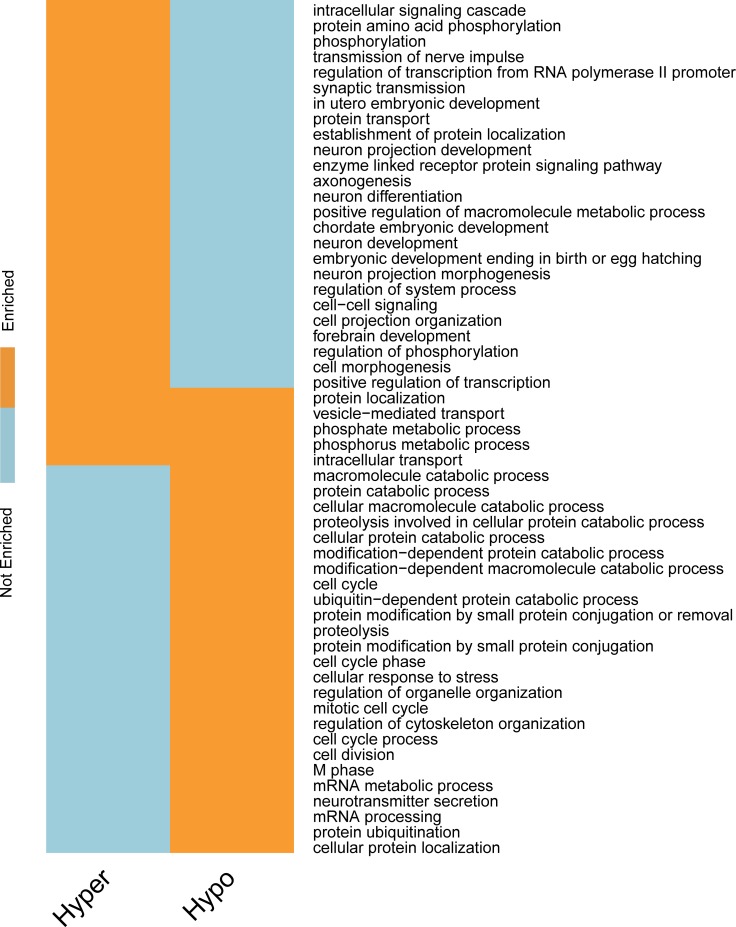
Biological processes regulated by FTO. We show here a binary map depicting the GO biological process (BP) categories enriched in m^6^A-driven genes identified in KD-FTO experiment. The enrichment analysis is conducted for the hyper and hypo m^6^A-driven genes respectively using DAVID. The hyper FTO targeted m^6^A-driven genes are closely link to synaptic transmission and cell-cell signaling, which is consistent with previous research. And we also find several other significant biological processes and genes regulated by m^6^A such as embryonic development and neuron differentiation. This result demonstrates that m^6^A-Driver can identify biological functionally significant m^6^A-driven genes.

**Fig 9:**
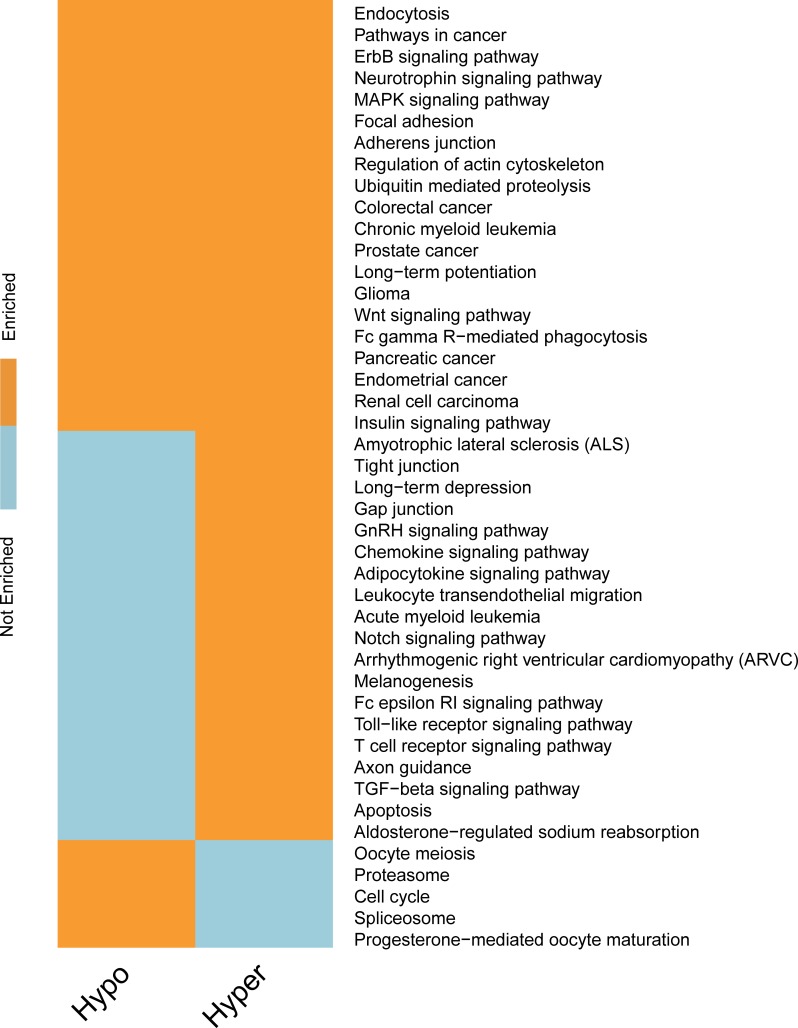
Pathways regulated by FTO. We show here a binary map depicting the KEGG categories most enriched in mDrGenes for KD-FTO dataset. The enrichment analysis is done to the hyper and hypo m^6^A-driven genes respectively using DAVID. The m^6^A-driven genes are significantly enriched in cancer related pathway and some specific cancer such as chronic myeloid leukemia and Glioma which suggests RNA methylation may play a role in cancer.

**Fig 10:**
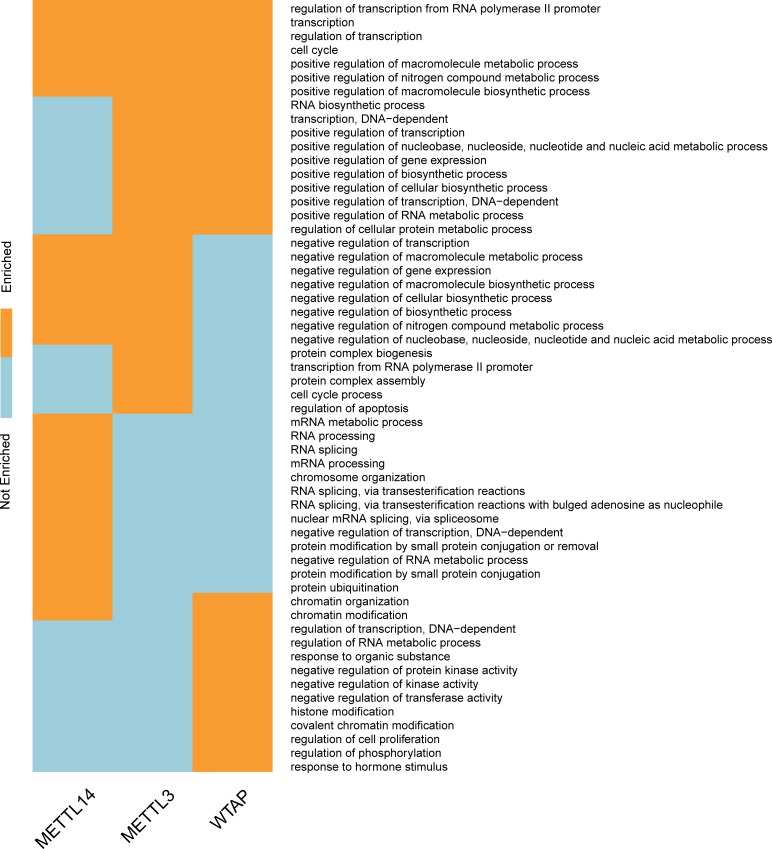
Biological processes regulated by methyltransferase complex. We show here a binary map depicting the GO biological process (BP) categories most enriched in mDrGenes identified in KD-METTL3, KD-METTL14 and KD-WTAP using DAVID. The 3 components of RNA methylation complex target different biological processes, indicating that different methylation enzymes may influence different biological processes via driving different genes.

**Fig 11:**
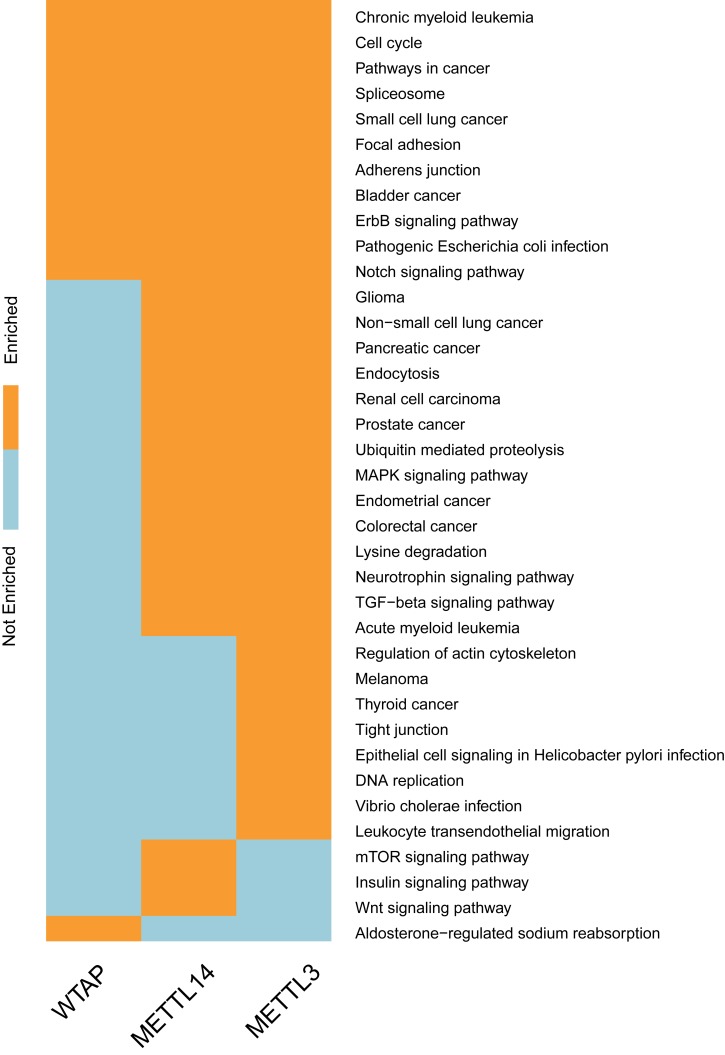
Pathways regulated by methyltransferase complex. We show here a binary map depicting the KEGG categories most enriched in mDrGenes identified in KD-METTL3, KD-METTL14 and KD-WTAP using DAVID. There are significant overlapping pathways between the three enzymes targeted m^6^A-driven genes and also enzyme specific functions. Two important consistent pathways are cancers and splicing which indicates m^6^A may regulate these pathways through m^6^A-driven genes.

Functional enrichment analyses were carried out on the 4 mDrNets to help reveal the biological processes regulated by the 4 enzymes at epitranscriptomic layer of gene regulation. The results obtained using DAVID reveal a significant enrichment of multiple m^6^A-related pathways annotated by either the Kyoto Encyclopedia of Genes and Genomes (KEGG) [53] or Gene Ontology (GO) biological process (BP) domains (Figs 8–11).

For KD-FTO, since FTO is a demethylation enzyme, we expected to observe mainly hyper-methylation. However, m^6^A-Driver did report several hypo-methylation mDrGenes, suggesting a potentially direct or indirect mode of FTO regulation to also enhance m^6^A. We next examined the functional relationship between the hyper- and hypo-methylated genes and found that there was little overlapping between their enriched functions (Fig 8 and Fig 9, see S6 Fig and S7 Fig for detail). To keep consistency with paper [27], in which the KD-FTO data is published, we adopt the whole reference genome as the control data set of enrichment analysis. The hyper-methylated mDrGenes are clearly linked closely to neurological processes and neuro signaling pathways. Several significantly enriched terms annotated by GO BP are synapse and neuron signaling transmission (132 hyper mDrGenes, p_Bonferroni_ = 1.79×10^−18^), synaptic transmission (43 hyper mDrGenes, p_Bonferroni_ = 1.77×10^−13^), transmission of nerve impulse (50 genes in hyper mDrGenes, p_Bonferroni_ = 5.58×10^−14^).They are also likely associated with neuron differentiation (65 hyper mDrGenes, p_Bonferroni_ = 1.81×10^−11^) and neuron development (53 hyper mDrGenes, p_Bonferroni_ = 3.00×10^−11^) as well as embryonic development (54 hyper mDrGenes in utero embryonic development, p_Bonferroni_ = 2.31×10^−13^ and 67 hyper mDrGenes in chordate embryonic development, p_Bonferroni_ = 2.42×10^−11^), which may be another proof of RNA methylation involved in steering stem cell pluripotency [34–36]. In contrast, the hypo-methylated mDrGenes are more related to metabolic processes (79 hypo mDrGenes in protein catabolic process, p_Bonferroni_ = 2.41×10^−18^ and 85 hypo mDrGenes in macromolecule catabolic process, p_Bonferroni_ = 1.88×10^−17^) and cell cycle (65 hypo mDrGenes, p_Bonferroni_ = 9.29×10^−12^). In addition, the hypo mDrGenes are enriched in Spliceosome (17 hypo mDrGenes, p_Bonferroni_ = 8.25×10^−4^), which is also a KEGG term enriched in KD-METTL3, KD-METTL14 and KD-WTAP data (Fig 11), implicating a potential role of m^6^A in mRNA splicing. Note that WTAP itself is also splicing factor. However, this result suggests that WTAP might also regulate splicing in an m^6^A dependent fashion. Taken together, our predicted mDrGenes confirm the demethylation role of FTO but may suggest a direct or indirect role of FTO in promoting m^6^A. Functional enrichment suggests that these two modes of FTO function are involved in distinct biological processes and pathways.

Another interesting finding is that both hyper and hypo mDrGenes are enriched in cancer related pathways including Chronic myeloid leukemia (20 hyper mDrGenes, p_Bonferroni_ = 3.27×10^−6^; 12 hypo mDrGenes, p_Bonferroni_ = 2.12×10^−3^) and Glioma (16 hyper mDrGenes, p_Bonferroni_ = 8.01×10^−5^; 12 hypo mDrGenes, p_Bonferroni_ = 4.86×10^−4^) (Fig 9, see S7 Fig for detailed information).

As FTO-KD data is extracted from mouse brain, so we also do enrichment analysis using the brain tissue specific expressed genes as control data to check whether the pathways we find above are really influenced by m^6^A methylation or by tissue specific expression. Brain tissue specific expressed genes are defined here as genes who have a RPKM value over 1 in at least half of the input samples, including treated and untreated ones. As is shown in S8 and S9 Figs, the results are similar to make the whole reference genome as control data set, but the enriched pathways get a bigger q-value due to the size reducing of control datasets, such as synaptic transmission (43 hyper mDrGenes, p_Bonferroni_ = 1.09×10^−5^), transmission of nerve impulse (50 genes in hyper mDrGenes, p_Bonferroni_ = 1.98×10^−5^), neuron differentiation (65 hyper mDrGenes, p_Bonferroni_ = 1.89×10^−8^) and Pathways in cancer (64 hyper mDrGenes, p_Bonferroni_ = 5.12×10^−6^; 40 hypo mDrGenes, p_Bonferroni_ = 3.51×10^−2^). These results show that the mDrGenes enriched pathways are really influenced by m^6^A methylation.

To further study the dynamics of m^6^A methylation, we applied the enrichment analysis to mDrGenes predicted in KD-METTL3, KD-METTL14 and KD-WTAP and compared the similarity and difference of their enriched GO biological processes (Fig 10, and also see S10 Fig for more details) and KEGG pathways (Fig 11, and also see S11 Fig for more information). In this case, we chose to perform enrichment on all mDrGenes in these three datasets instead of analyzing hyper- and hypo-methylated mDrGenes separately, because there are little hyper mDrGenes in these 3 datasets. There are significant overlapping biological processes among these 3 sets of mDrGenes, but also exist enzyme specific functions (Fig 10**)**. The common biological processes include cell cycle (143 mDrGenes in KD-METTL3, p_Bonferroni_ = 2.52×10^−20^, 123 mDrGenes in KD-METTL14, p_Bonferroni_ = 6.71×10^−15^, 51 mDrGenes in KD-WTAP, p_Bonferroni_ = 2.35×10^−10^), regulation of transcription (324 mDrGenes in KD-METTL3, p_Bonferroni_ = 7.76×10^−16^, 315 mDrGenes in KD-METTL14, p_Bonferroni_ = 2.37×10^−19^, 103 mDrGenes in KD-WTAP, p_Bonferroni_ = 1.88×10^−7^) and positive regulation of molecular metabolism, e.g., positive regulation of macromolecule metabolic process (137 mDrGenes in KD-METTL3, p_Bonferroni_ = 5.19×10^−14^, 126 mDrGenes in KD-METTL14, p_Bonferroni_ = 8.12×10^−13^, 55 mDrGenes in KD-WTAP, p_Bonferroni_ = 9.27×10^−11^).

What is interesting is that the overlapping functions between METTL3 associated mDrGenes and WTAP associated mDrGenes are positive regulations of metabolism and gene expression. In contrast, the overlapping functions between METTL3 targeted mDrGenes genes and METTL14 mDrGenes are mainly negative regulation of metabolism and gene expression. The METTL14 mDrGenes are also strongly enriched in splicing, especially, e.g., RNA splicing (76 mDrGenes, p_Bonferroni_ = 2.71×10^−22^), RNA splicing, via transesterification reactions (43 mDrGenes, p_Bonferroni_ = 1.38×10^−13^) and RNA splicing, via transesterification reactions with bulged adenosine as nucleophile (43 mDrGenes, p_Bonferroni_ = 1.38×10^−13^). It is consistent with that the most enriched motif in KD-METTL14 is similar to the binding motifs of SRSF1 and SRSF9, two factors involved in alternative splicing. These suggest a potential role of METTL14 in regulating splicing via m^6^A. In contrast, WTAP mDrGenes are enriched specifically in chromatin modification (26 genes, p_Bonferroni_ = 1.46×10^−8^), whereas METTL3 mDrGenes may influence the development of protein complex, e.g., protein complex assembly (96 mDrGenes, p_Bonferroni_ = 1.86×10^−14^) and protein complex biogenesis (96 mDrGenes, p_Bonferroni_ = 1.86×10^−14^).

Comparison of top KEGG pathways enriched in the three mDrGenes sets also revealed common as well as methylase specific functions (see Fig 11). Particularly, the consensus functions include cell cycle (38 mDrGenes in KD-METTL3, p_Bonferroni_ = 2.19×10^−11^, 27 mDrGenes in KD-METTL14, p_Bonferroni_ = 8.64×10^−7^, 16 mDrGenes in KD-WTAP, p_Bonferroni_ = 1.08×10^−5^), spliceosome (34 mDrGenes in KD-METTL3, p_Bonferroni_ = 8.9×10^−9^, 26 mDrGenes in KD-METTL14, p_Bonferroni_ = 3.51×10^−6^, 14 mDrGenes in KD-WTAP, p_Bonferroni_ = 2.06×10^−4^) and pathway in cancer (66 mDrGenes in KD-METTL3, p_Bonferroni_ = 2.68×10^−10^, 57 mDrGenes in KD-METTL14, p_Bonferroni_ = 8.58×10^−10^, 30 mDrGenes in KD-WTAP, p_Bonferroni_ = 5.46×10^−7^) especially Chronic myeloid leukemia (28 mDrGenes in KD-METTL3, p_Bonferroni_ = 8.66×10^−11^, 21 mDrGenes in KD-METTL14, p_Bonferroni_ = 2.52×10^−7^, 12 mDrGenes in KD-WTAP, p_Bonferroni_ = 2.58×10^−5^). The significant overlapping pathways between METTL3 and METTL14 include Glioma (20 mDrGenes in KD-METTL3, p_Bonferroni_ = 1.37×10^−6^, 13 mDrGenes in KD-METTL14, p_Bonferroni_ = 1.85×10^−3^), suggesting that these mDrGenes may be used as biomarkers of glioma. We also notice that METTL3 mDrGenes are specifically enriched in Melanoma (18 mDrGenes, p_Bonferroni_ = 1.32×10^−4^). A recent study have demonstrated that mutations within intron 8 of FTO leads to increased melanoma risk [29], suggesting a link between m^6^A and melanoma.

To help reveal the pathways potentially relevant to different modes of m^6^A functions, we checked the overlaps between the enriched pathways in hyper and hypo mDrGenes in KD-FTO and mDrGenes in KD-METTL3, KD-METTL14 and KD-WTAP (S12 and S13 Figs). All 5 groups of mDrGenes are enriched in cell cycle and pathways in cancer, including especially Chronic myeloid leukemia. This further suggests that m^6^A is related to cancer. The overlapping pathways between the hyper-mDrGenes in KD-FTO and those in METTL3/METTL14 are mainly related to transcription including regulation of transcription and regulation of gene expression. Indeed, it has been shown that m^6^A recruits YTHDF2 protein to regulate mRNA stability [54]. In contrast, the overlapping pathways between the hypo-mDrGenes in KD-FTO and those in METTL3/METTL14 are related RNA splicing. Interestingly, nuclear m^6^A-binding protein YTHDC1 is shown to promote exon inclusion of targeted mRNAs through facilitating mRNA binding of splicing factor SRSF3 [55,56].

**Fig 12:**
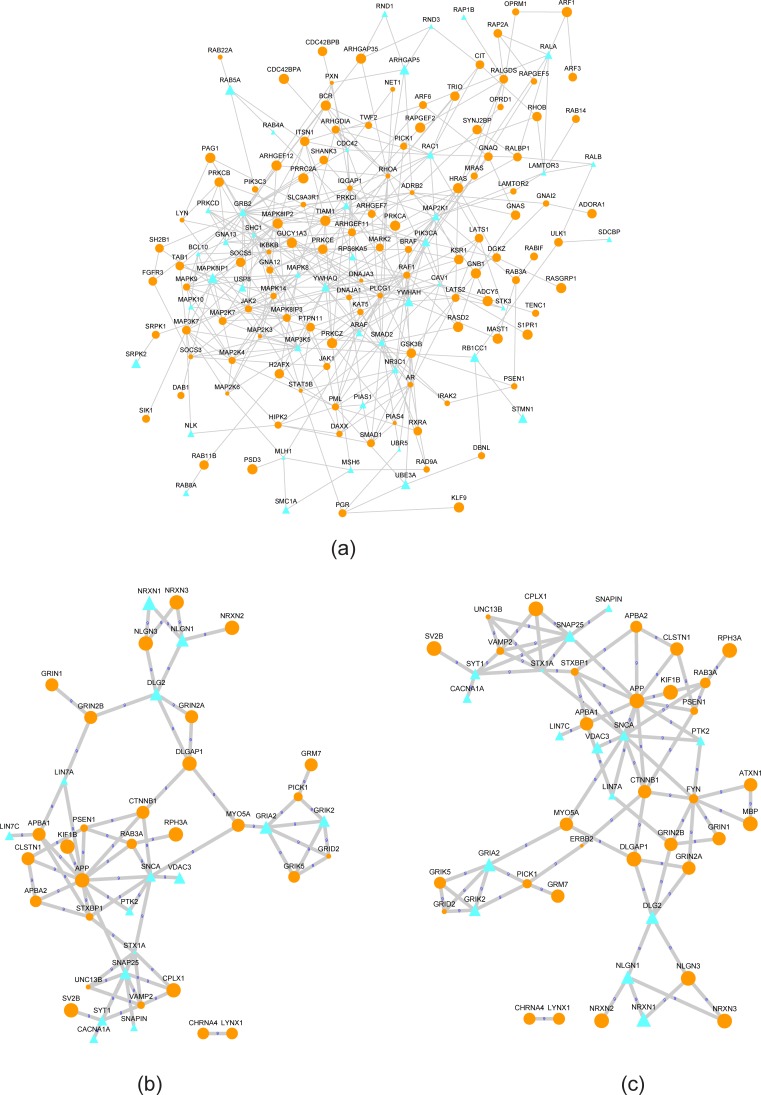
Subnetworks of mDrNet in KD-FTO. (a) Sub-mDrNet associated with intracellular signaling cascade BP category. (b) Sub-mDrNet associated with synaptic transmission BP category. (c) Sub-mDrNet associated with transmission of nerve impulse BP category. The orange circle nodes denote hyper mDrGenes and the cyan triangle nodes denote hypo mDrGenes. The node size and edge width are corresponding to the frequency that they appear in different replicates sets. The number labeled on the edge means the times that it reappears in all RS sets. mDrGenes in the same biological process interact with each other closely, which is consistent with our hypothesis. For UNC13B, STX1A and GRID2, although they are not predicted as differential m^6^A genes in all RS, m^6^A-Driver still identify them as mDrGenes, because they have significant biological functions.

We further examined the mDrNets and their subnetworks associated with the enriched biological processes. Several sub-mDrNets for KD-FTO including intracellular signaling cascade, synaptic transmission BP category, and transmission of nerve impulse, are shown in Fig 12. They are consistent with our hypothesis that mDrGenes in the same biological process are interacted with each other closely. That is also the reason why m^6^A-Driver can identify mDrGenes that might not be consistently identified in most RSs due to the biological variance, but have significant biological functions. This underscores the advantage of m^6^A-Driver in addressing variance among different replicates for predicting mDrGenes.

**Fig 13:**
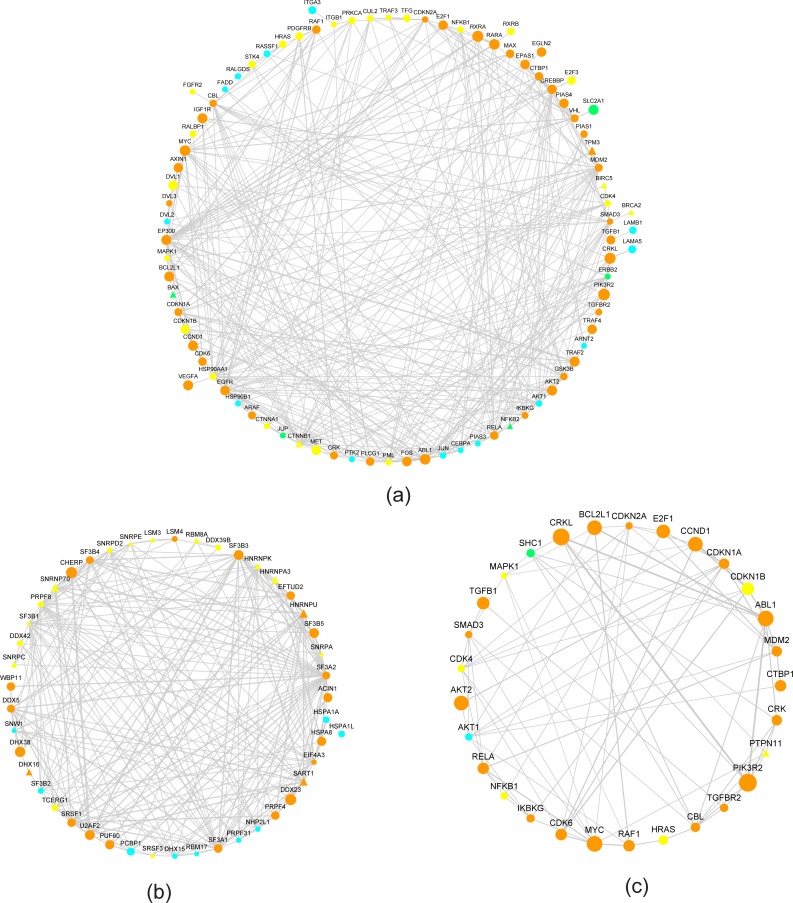
Sub-mDrNet of KD-METTL3, KD-METTL14 and KD-WTAP. (a) Sub-mDrNet associated with Pathways in cancer. (b) Sub-mDrNet associated with Spliceosome. (c) Sub-mDrNet associated with chronic myeloid leukemia. The circle nodes are hypo mDrGenes and the triangle ones are hyper mDrGene. The orange nodes denote mDrGenes reappearing in at last two consensus networks. The yellow nodes denote mDrGenes specifically from KD-METTL3 mDrNet. The cyan nodes denote KD-METTL14 specific mDrGenes and the green ones denote WTAP specific mDrGenes. The node size and the edge width represent the frequency that they reappear in different replicates sets (RSs). Though enriched in the same pathways but the target mDrGenes are not all the same for the 3 methylation enzymes. Mean that, the 3 methylation enzymes may drive different genes to influence the same pathway.

For KD-METTL3, KD-METTL14 and KD-WTAP, since they form the m^6^A methyltransferase complex, we integrated the 3 mDrNets and examined the subnetworks associated with the enriched pathways (Fig 13). Similar to KD-FTO, the mDrGenes enriched in the same pathway are closely connected and many mDrGenes undetected as differential m^6^A genes in all RSs are also identified. What is also interesting to notice is that the enriched pathways common in three datasets could be resulted from very different mDrGenes for each dataset, suggesting the 3 m^6^A methylases collaboratively regulate the same pathway through different mechanisms.

Because many m^6^A sites are detected in 3’UTRs that also contain microRNA binding sites. It will be helpful to further examine if the mDrGenes are also enriched in certain microRNA with significant functions. To test this, we download the microRNA-target information from miRTarBase, a database curates experimentally validated microRNA-target interactions [57]. Then we performed Fisher’s test to test whether the m^6^A driven genes are enriched in targets of certain microRNA families. Interestingly, although most mDrGenes are targeted by microRNAs (60% in KD-FTO, 96% in KD-METTL3, 97% in KD-METTL14 and 97% in KD-WTAP), not many microRNAs have targets enriched in mDrGenes. For KD-FTO, there are only 2 microRNAs have p-value < 0.05, and for KD-METTL3, KD-METTL14 and KD-WTAP, there are only 1, 1, 0, separately (Table 2). The information of all targeted mDrGenes by microRNA is included in supplementary material (S1–S4 Texts).

## Discussion

The MeRIP-seq technology significantly advances the study of m^6^A, enabling profiling m^6^A methytranscriptome for specific cell conditions. However, existing algorithms focus mostly on predicting m^6^A sites from MeRIP-seq data. Although they are powerful tools for MeRIP-seq data analysis, they cannot directly assess the functional importance of these sites and associated genes. To address this shortcoming, we proposed in this paper m^6^A-Driver, a novel algorithm for detecting m^6^A-driven genes and their interaction network. m^6^A-Driver utilizes protein-protein interaction networks to identify functional meaningful differentially m^6^A methylated genes and overcomes the biases in predicting functional enrichment of sites due to different sample replicates. The comparison on the *p*-values of top enriched biological processes in the prediction results of m^6^A-Driver and exomePeak demonstrates that m^6^A-Driver could identify mDrGenes that are more functional relevant. In terms of the algorithm, comparison with VarWalker, an algorithm for predicting mutation driver genes, shows that m^6^A-Driver is computationally more efficient and can produce topological and biologically more robust predictions. Furthermore, m^6^A-Driver generates a condition-specific m^6^A-driven network that reveals the detailed functional circuitry underlying the biological condition.

The results on the FTO, METTL3, METTL14 and WTAP knockdown data demonstrated that m^6^A-Driver can address the sample bias in MeRIP-Seq data and identify functional relevant mDrGenes in a robust and efficient fashion. m^6^A-Driver predicted several significant biological progresses and pathways associated with each knockdown dataset and constructed four mDrNet separately regulated by the four m^6^A (de)methylases. Functional enrichment analysis across the four networks showed the involvement of m^6^A in a diverse processes and pathways including regulating cancer, transcription, and slicing, many of which have been reported in the literature. The presented results in this paper demonstrate that m^6^A-Driver is an effective and reliable approach to identify functionally relevant m^6^A-driven genes and networks from MeRIP-Seq data.

We want to point out that the inherent technical limitations of MeRIP-Seq can lead to increased false positives in the predicted mDrGenes and their functional networks. As pointed out earlier in the paper, the sample bias and the impact of library “size factor” from different conditions can negatively impact the quality of the data. How to normalize MeRIP-Seq samples from different conditions is still an open topic that requires additional research. Many useful normalization methods such as the "geometric mean" approach proposed in DESeq can provide valuable guidance. Nevertheless, the results from this and other papers showed that with careful processing and modeling of the current MeRIP-Seq data, many important functions of m^6^A can be predicted. With the continuing improvement of the MeRIP-Seq and related technologies, we expect that m^6^A-Driver should produce increasingly more accurate predictions. In the current m^6^A-Driver algorithm, identification of mDrGenes relies on a reference network and a threshold to determine the candidate genes. So far, only PPI network is considered, and no reference networks for noncoding RNAs are included. As m^6^A is also prevalent in long noncoding RNAs (lncRNAs), constructing a noncoding RNA interaction network and extending m^6^A-Driver algorithm to lncRNAs will enable the study of m^6^A associated functions in lncRNAs. As a future work, we can use the score in step 4 to help construct the consensus network. For a DmMGs network of a biological replicate sets built by step 3, we can use peak calling score to transfer the network to a weighted network. The weight of an edge in the network could be calculated as $w=\frac{-\mathrm{lg}(x)-\mathrm{lg}(y)}{2}$, where *x* and *y* denote the peak calling FDR (adjusted p-value) of the two gene nodes. Then all edges of the DmMGs networks are pooled together. For each edge, we can calculate the consensus score as $s=\sum_{i=1}^{m}w_{i}$, where *m* is the number of replicate sets and *w*_*i*_ is the weight of the edge in network *i*. The final consensus m^6^A driven gene network can be built by setting a threshold on *s* according to its distribution or the scale of the consensus network we need.

## Materials and Methods

### Datasets

FTO knockdown (KD-FTO) dataset is a MeRIP-Seq data [27] from the wild-type littermate as well as FTO knockdown mice samples. There are 9 sets of biological replicates in this cohort, and each biological replicate set (RS) contains two IP samples from a FTO knockdown mouse and the wild-type littermate, respectively, as well as two corresponding input samples from the two mice samples. In the original work [27], Hess et al. demonstrated that FTO-knockdown mice have impaired dopamine release, reduced dopaminergic receptor responses and an altered locomotor response to cocaine, which are related to specific m^6^A mRNA demethylation regulated by FTO.

METTL3, METTL14 and WTAP knockdown MeRIP-Seq datasets are from a recent study which reveals that m^6^A regulates mRNA stability [44]. Each of the three cohorts consists of 4 RSs, each of which contains two IP samples from gene knockdown (treated) HeLa cells and untreated HeLa cells, respectively, and two corresponding input samples. The study revealed that knockdown of METTL3 led to decrease the binding of YTHDF2 to its targets, and increase the stability of its target RNAs similar to that of YTHDF2 knockdown.

The reference network, PPI network, is built from the most recent version of PPI data from BioGRID (release 3.4.128, compiled on August 25th, 2015) [45]. Based on the binary interactions, we removed the isolated and self-interactions proteins to establish a PPI network with a total of 16,062 proteins and 152,676 interactions.

### Prediction of m^6^A-driven genes and the network using the m^6^A-Driver algorithm

m^6^A-Driver predicts mDrGenes and mDrNet from MeRIP-Seq data, where mDrGene is a gene whose mRNA harbors at least one DmM site in a biological context of interest and whose function is relevant to the context. m^6^A-Driver first divides the MeRIP-Seq data into several RSs, each containing 2 paired samples, an IP sample paired with an input sample under the treated condition and another pair under the untreated condition. Then, m^6^A-Driver predicts mDrGenes and mDrNet by the following four steps. The workflow is shown in Fig 1.

#### Step 1

RS-specific prediction of DmMG using exomePeak. This step is designed to predict DmMGs for each RS. A gene is identified as a DmMG if its mRNA harbors at least one hyper- or hypo-methylated m^6^A site in treated samples compared with untreated samples. The DmM sites of a RS are detected by the exomePeak R package [38], which predicts the transcriptome-wide m^6^A hyper- or hypo-methylated sites from the treated and untreated MeRIP-Seq datasets. At the end of this step, a set of DmMGs are obtained for each RS.

#### Step 2

Prediction of RS-specific candidate DmMGs with the Random Walk with Restart algorithm. The goal of this step is to predict potential DmMGs that not detected in a RS due to the biological variance. RWR simulates a random walker from either a seed node or a set of seed nodes, and moves to its immediate neighbors randomly at each step until it reaches a stable status. In the end, all the nodes in the graph are ranked by the probability of the random walker reaching this node, which denotes the degree of closeness to the seed node(s). Given a connected graph ***G*** with *N* nodes, RWR can be formally described as follows:

$$p^{t+1}=(1-\lambda )Mp^{t}+\lambda p^{0}$$ (1)

where ***p***^*t*^ is an *N*×1 vector whose *i*th element represents the probability of the walker traversing to node *i* at step *t*, ***p***^0^ is the *N*×1 initial probability vector, ***M*** is an *N*×*N* transition matrix of the graph, which is the column-normalized adjacency matrix for ***G***, and *λ* is a fixed parameter, which denotes the restarting probability at a given time step (*λ* = 0.5 in this study). Generally, if assuming that there are *k* initial genes from which the walker would start with an equal probability, the initial nodes will have a probability of 1 / *k* and the remaining nodes will have a probability 0 in ***p***^0^, meaning that $p^{0}=\left\{p_{\mu }^{0}\right\}=\{\begin{array}{c} 1/k,\;\text{start node}\\ 0,\;\text{otherwise} \end{array}$, where *μ* = 1,2,⋯,*n*. Then, ***p***^*t*^ is updated according to (1) iteratively until the difference between ***p***^*t*^ and ***p***^*t*+1^ is below a predefined threshold (10^−6^ in this work).

For each RS, we iteratively take each DmMG as the starting node, performing RWR to prioritize candidate DmMGs. According to the probability that the walker would reach the node after the RWR enters the stable state, nodes ranked top 10 are held as candidate DmMGs. Previous studies suggested various ways to select candidate nodes including using the most accessible node (i.e., top 1) [58, 59], top 5 [60], top 10 [61,62], top 20 [62], and top 100 nodes [63], but no consensus rules have been proposed. In this work, we retained the top 10 accessible nodes to keep a balanced tradeoff between choosing too few informative genes (e.g. top 1) and too many irrelevant genes [46]. The threshold, top 10, is only an empirical primary filter of nodes to select only the most highly accessible nodes from the starting node (DmMG); the candidate DmMGs will be further assessed by their topological and biological significance in the next step.

#### Step 3

Topological and functional significance-based evaluation of the candidate DmMGs. The goal of this step is to extract functionally and topologically significant candidate DmMGs from the PPI network. To evaluate the topological significance of the candidate nodes in the PPI network, Jia et al. proposed a strategy that utilizes randomized PPI networks that maintain the topological characteristics of the original network (e.g., degree of each node) to estimate whether the candidate target genes defined as gene interact with target gene (i.e., DmMG identified in step 1 in this paper) are identified by chance [46]. To this end, 100 random PPI networks, each of which preserves the degree distribution of the original PPI network, are first generated through the switching algorithm proposed by Moli et al. [64]. Then, RWR is performed, using each of DmMG as a starting node, in each of the 100 random networks and the top 10 nodes with the highest probabilities are extracted. Finally, for each DmMG, the 10 candidate nodes in the original network, *g*_1_,*g*_2_,⋯,*g*_10_, are assessed by computing an empirical *p*-value: $p=\frac{\#\{\pi (g_{\nu })\}}{100}$, where *π*(*g*_*ν*_) is a random network in which *g*_*ν*_(*ν* = 1,2,⋯,10), is found as the top 10 candidate genes after the restart random walk starting from the target DmMG. The empirical *p*-value is calculated as the probability of a candidate DmMG to be randomly selected. According to the study of Jia et al., the candidate DmMGs with *p* < 0.05 should be retained as the significant candidate DmMGs.

Note that the random PPI network maintains only the degree distribution of the original network, indicating that the degree is the only property of nodes in the random network. Since the rank of a gene in a random network is determined by its degree and the initial node's degree, it is possible that this degree-based filter of candidate DmMGs may remove functional significant genes by mistake. Meanwhile, the randomization-based strategy may remove different candidate genes for the same DmMG when adopting different sets of random PPI networks. In addition, performing RWR for each DmMG of each sample in each of the 100 networks will take a long time.

To address these issues, we combine the degree distributions of top 10 genes prioritized by starting nodes with different degree in random networks and use the shortest path between candidate DmMG and DmMG to assess candidate DmMGs. Although PPI network consists of 16,062 nodes, these nodes only hold 312 kinds of degree *d*_*i*_(*d*_*i*_∈[1,1975], *i* = 1,2,⋯,312). For each kind of degree, we randomly selected a node with degree *d*_*i*_ as the starting node, denoted as *Seed*_*i*_(*i* = 1,2,⋯,312), to initiate the random walk in each random network and retained the top 10 nodes with the highest probabilities. After assessing all the 100 random networks, a candidate DmMGs set ***S***_*j*_(*j* = 1,2,⋯,312) can be formed. The normal occurrence of gene with degree *d*_*i*_ in ***S***_*j*_ is calculated as: $freq_{ij}=\frac{\#\text{random occurrence}}{\#\text{PPI occurrence}}$ where "#random occurrence" is the counts of genes with degree *d*_*i*_ selected as top 10 across the 100 random networks, that is, the counts of genes with degree *d*_*i*_ in ***S***_*j*_, and "#PPI occurrence" is the counts of genes with degree *d*_*i*_ in PPI network. Finally, we compute an empirical *p*-value: $p_{ij}=\frac{freq_{{}_{ij}}}{100}$ for the gene with degree *d*_*i*_ in ***S***_*j*_, which indicates the probability of the gene to be randomly selected [46]. Moreover, to make the filter more robust, we formulate a function *p*_*ij*_ = *f*_*j*_(*d*_*i*_) to estimate the empirical *p*-value of gene with degree *d*_*i*_ in ***S***_*j*_, and then solve it using the locfit algorithm [65].

**Fig 14:**
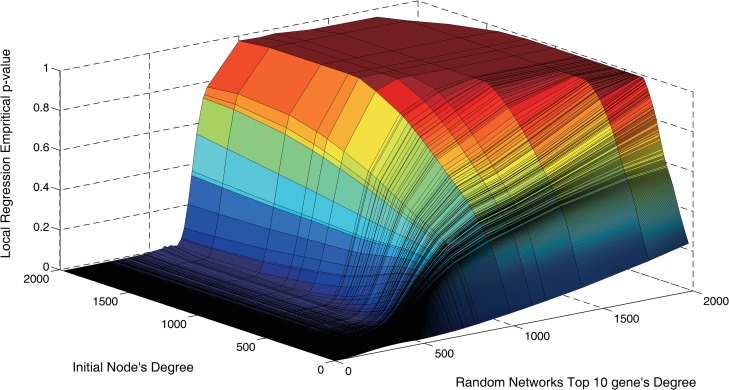
The influence of nodes degree on the empirical *p*-value [46]. The top 10 genes of a random network denote the gene that rank top 10 in the random network initiated by the starting node with a specific degree. We can see that the *p*-value monotonically increases along with the increase of top 10 genes' degree and the growth rate is enhanced when initial node's degree is larger.

Fig 14 shows the empirical *p*-value as a function of the degree, which is used to select top 10 genes in the random network initiated by the starting nodes with different degree. As is shown that the empirical *p*-value monotonically increases along with the increase of top 10 genes' degree and the rate of increase is higher if the initial node's degree is larger. This implies that filtering candidate genes based only on the empirical *p*-values could remove candidate genes with biological significance.

To filter the candidate genes of a DmMG by both their topological and functional significance, we first compute the empirical *p*-value of candidate DmMGs. For a candidate DmMG with degree *d*_*i*_, the *p*-values calculated by the regression function, *p*_*ij*_ = *f*_*j*_(*d*_*i*_). Then, we calculate the shortest path *L* between candidate DmMGs and the corresponding DmMG in PPI network, which assesses the functional significance of the candidate DmMG. Finally, we retain candidate DmMGs with *p* < 0.05 or *L* = 1 as significant candidate DmMGs. The procedure is iteratively performed to candidate genes of each DmMG in each RS.

#### Step 4

Construction of a consensus m^6^A-driven gene network. The goal of this step is to extract mDrGenes from DmMGs identified in each RS. After detecting DmMGs and their candidate genes in each RS, all significant interactions from different RS are pooled together, forming a universal candidate edges pool. Furthermore, we required both proteins involved in an interaction to be encoded by DmMGs, meaning that a pair of DmMGs and its candidate genes in the pool could be either identified in the same RS or in different RS set. We extracted edges that consistently occur in every RS, and the DmMGs involved in the edges were identified as m^6^A-driven genes. Then all the eligible edges constitute the consensus mDrNet. The selected mDrGenes are DmMGs identified in at least one RS, which means they contain the information from original data, but they are not required to reappear in every RS to avoid sample bias. Also, mDrGenes are identified by consensus reappearance across all RS to enhance their reliability. Moreover, mDrGenes are closely interacting in the PPI network, implying significant functions.

## Supporting Information

S1 Fig ROC-like curves of three mrthylation enzymes knockdown data sets. The false positive rate (FPR) is the ratio of pseudo DMSs to all m^6^A methylation sites and the reported true positive rate (RTPR) is the ratio of real DMSs to all m^6^A methylation sites. The black line is the line of y = x.
(TIFF)

S2 Fig Consensus network in KD-FTO dataset. The network consists of m^6^A-driven genes identified in KD-FTO dataset. We can see that they are closely interacted with each other in the network which indicates that m^6^A-driven genes regulated by FTO are functionally relevant.
(TIFF)

S3 Fig Consensus network in KD-METTL3 dataset. The network consists of m^6^A-driven genes identified in KD-METTL3 dataset. We can see that they are closely interacted with each other in the network which indicates that m^6^A-driven genes regulated by METTL3 are functionally relevant.
(TIFF)

S4 Fig Consensus network in KD-METTL14 dataset. The network consists of m^6^A-driven genes identified in KD-METTL14 dataset. We can see that they are closely interacted with each other in the network which indicates that m^6^A-driven genes regulated by METTL14 are functionally relevant.
(TIFF)

S5 Fig Consensus network in KD-WTAP dataset. The network consists of m^6^A-driven genes identified in KD-WTAP dataset. We can see that they are closely interacted with each other in the network which indicates that m^6^A-driven genes regulated by WTAP are functionally relevant.
(TIFF)

S6 Fig Biological processes regulated by FTO. We show here a heat map depicting the GO biological process (BP) categories most enriched in m^6^A-driven genes identified in KD-FTO dataset. The enrichment analysis is conducted for the hyper and hypo m^6^A-driven genes respectively using DAVID. The FTO targeted hyper m^6^A-driven genes closely link to synaptic transmission and cell-cell signaling. And we also find several other significant biological processes and genes regulated by m^6^A such as embryonic development and neuron differentiation. Thus demonstrates m^6^A-Driver could identify biological functionally significant m^6^A-driven genes.
(TIFF)

S7 Fig Pathways regulated by FTO. We show here a heat map depicting the KEGG categories most enriched in m^6^A-driven genes identified in KD-FTO dataset. The enrichment analysis is done to the hyperand hypo m^6^A-driven genes respectively using DAVID. The m^6^A-driven genes are significantly enriched in cancer related pathway and some specific cancer such as chronic myeloid leukemia and Glioma which suggest RNA methylation may play a role in cancer.
(TIFF)

S8 Fig Binary biological processes regulated by FTO using the brain tissue specific expressed genes as control data. We show here a binary map depicting the GO biological process (BP) categories enriched in m^6^A-driven genes identified in KD-FTO experiment. The enrichment analysis is conducted for the hyper and hypo m^6^A-driven genes respectively using DAVID and adopting the brain tissue specific expressed genes as control data.Brain tissue specific expressed genes are genes who have a RPKM value over 1 in at least half of the input samples, including treated and untreated ones.
(TIFF)

S9 Fig Binary pathways regulated by FTO using the brain tissue specific expressed genes as control data. We show here a binary map depicting the KEGG categories most enriched in mDrGenes for KD-FTO dataset. The enrichment analysis is conducted for the hyper and hypo m^6^A-driven genes respectively using DAVID and adopting the brain tissue specific expressed genes as control data. Brain tissue specific expressed genes are genes who have a RPKM value over 1 in at least half of the input samples, including treated and untreated ones.
(TIFF)

S10 Fig Biological processes regulated by methyltransferase complex. We show here a heat map depicting the GO biological process (BP) categories most enriched in m^6^A-diven genes identified in KD-METTL3, KD-METTL14 and KD-WTAP using DAVID. There are significant overlapping biological processes between the three enzymes targeted m^6^A-driven genes and also enzyme specific functions, suggesting that different methylation enzymes may influence different biological processes via driving different genes.
(TIFF)

S11 Fig Pathways regulated by methyltransferase complex. We show here a heat map depicting the KEGG categories most enriched in m^6^A-driven genes identified in KD-METTL3, KD-METTL14 and KD-WTAP using DAVID. There are significant overlapping pathways between the three enzymes targeted m^6^A-driven genes and also enzyme specific functions. Two important consistent pathways are cancers and splicing which indicates m^6^A may regulate these pathways through m^6^A-driven genes.
(TIFF)

S12 Fig Binary biological processes regulated by both FTO and methyltransferases. We show here a binary map depicting the GO biological process (BP) categories most enriched in mDrGenes identified in KD-FTO, KD-METTL3, KD-METTL14 and KD-WTAP using DAVID. Enriched BPs in KD-FTO are divided into hyper and hypo groups. We only keep BPs enriched in at least one group (hyper or hypo) of KD-FTO and enriched in at least 3 of the 5 groups to show significant overlap between the 5 group of BPs. The overlap BPs between FTO-hyper group and METTL3/METTL14 are mainly about transcription, regulation of transcription and regulation of gene expression. The overlap BPs between FTO-hypo group and METTL3/METTL14 are mainly about RNA splicing and protein modification. This indicates that the dynamic of m^6^A may regulate these biological processes in a direct or indirect way.
(TIFF)

S13 Fig Binary pathways regulated by both FTO and methyltransferases. We show here a binary map depicting the KEGG categories most enriched in mDrGenes identified in KD-FTO, KD-METTL3, KD-METTL14 and KD-WTAP using DAVID. Enriched pathways in KD-FTO are divided into hyper and hypo groups. We only keep pathways enriched in at least one group (hyper or hypo) of KD-FTO and enriched in at least 3 of the 5 groups to show significant overlap between the 5 group of pathways. All of the 5 group of mDrGenes are enriched in pathway in cancer, especially in Chronic myeloid leukemia. This further illustrate the dynamic of m^6^A is related to cancer. The overlap pathways between FTO-hypo group and METTL3/METTL14/WTAP are cell cycle and spliceosome. This is consistent with the result of BP enrichment analysis and confirm the relevance between dynamic m^6^A and splicing.
(TIFF)

S1 Text microRNAs targeting mDrGenes in KD-FTO. (TXT)

S2 Text microRNAs targeting mDrGenes in KD-METTL3. (TXT)

S3 Text microRNAs targeting mDrGenes in KD-METTL14. (TXT)

S4 Text microRNAs targeting mDrGenes in KD-WTAP. (TXT)
