# Supplementary figures and images for: m6A-Driver: Identifying Context-Specific mRNA m6A Methylation-Driven Gene Interaction Networks
Source: PLoS Comput Biol. 2016 Dec 27;12(12):e1005287. doi: 10.1371/journal.pcbi.1005287 (PMC5226821; doi:10.1371/journal.pcbi.1005287)

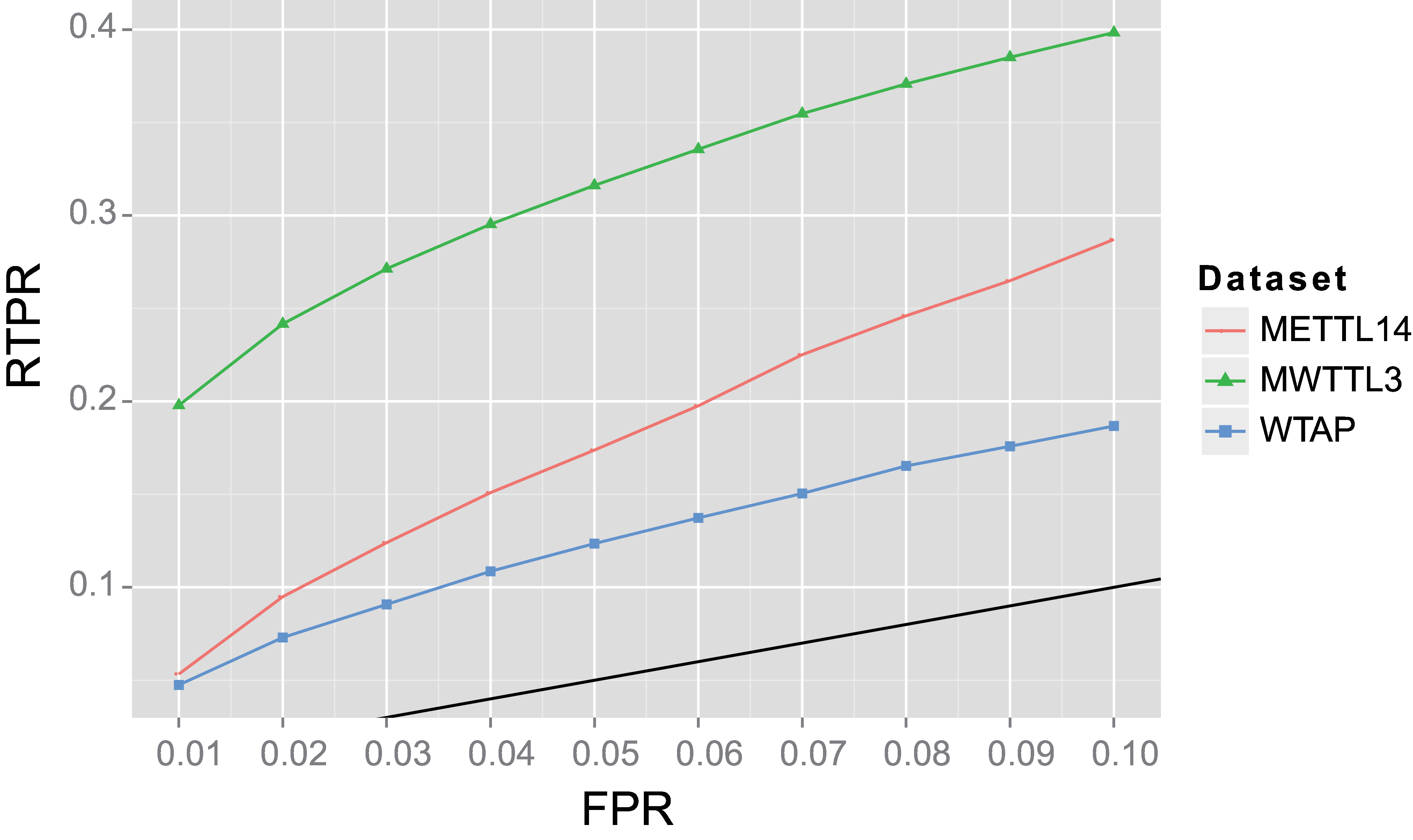

Supplement: S1 Fig — The false positive rate (FPR) is the ratio of pseudo DMSs to all m6A methylation sites and the reported true positive rate (RTPR) is the ratio of real DMSs to all m6A methylation sites. The black line is the line of y = x. (TIFF) [file pcbi.1005287.s001.tiff]

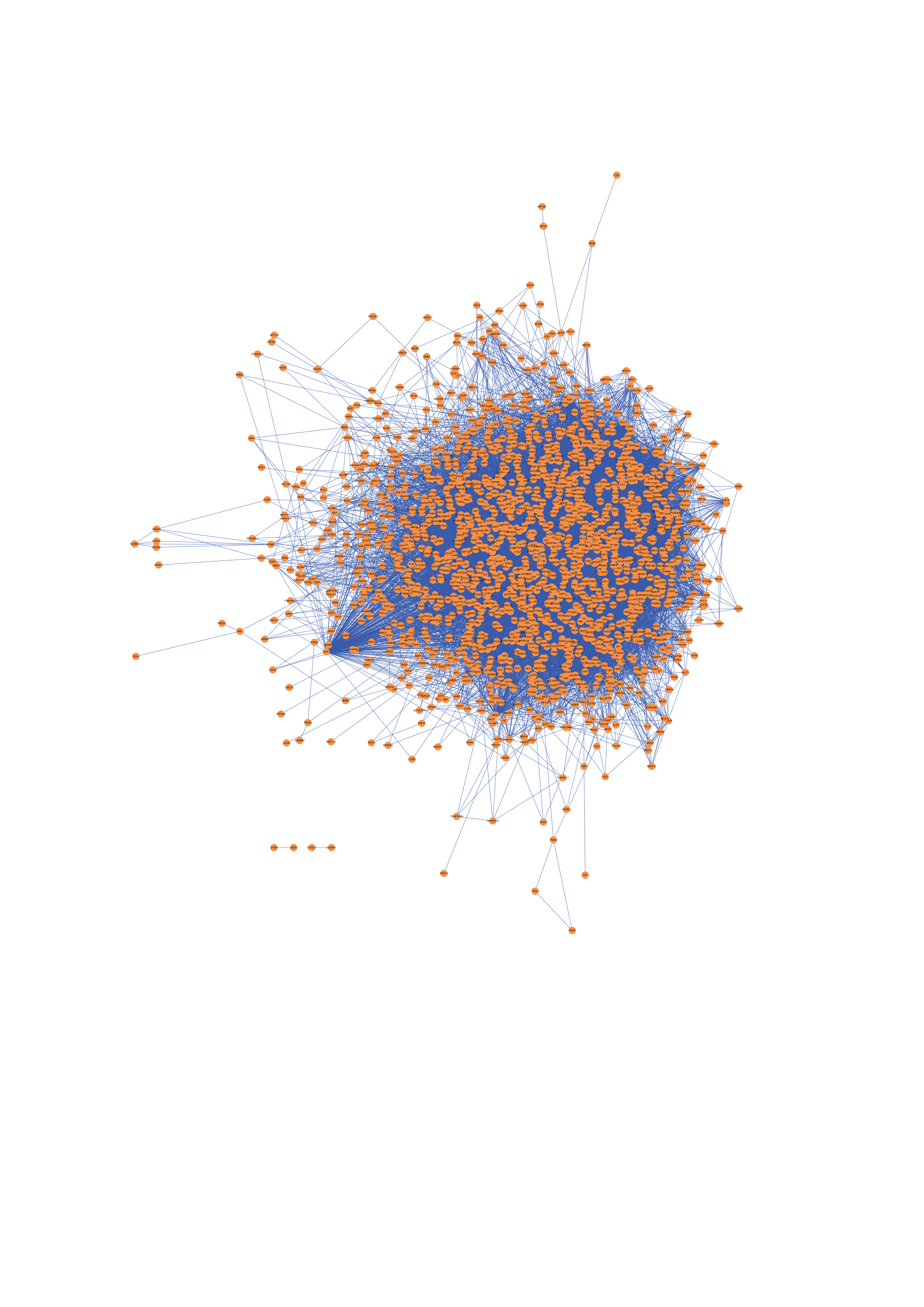

Supplement: S2 Fig — The network consists of m6A-driven genes identified in KD-FTO dataset. We can see that they are closely interacted with each other in the network which indicates that m6A-driven genes regulated by FTO are functionally relevant. (TIFF) [file pcbi.1005287.s002.tiff]

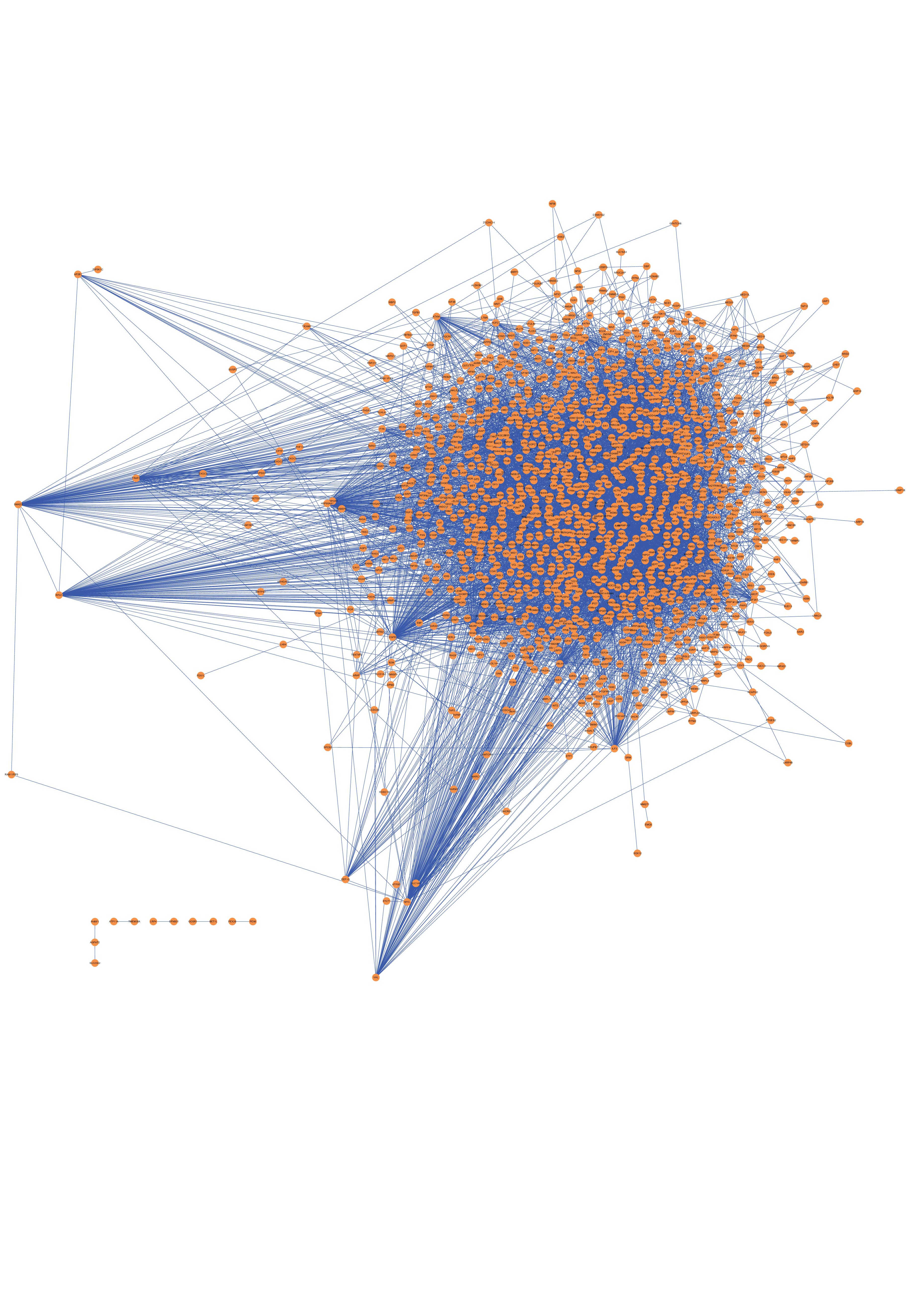

Supplement: S3 Fig — The network consists of m6A-driven genes identified in KD-METTL3 dataset. We can see that they are closely interacted with each other in the network which indicates that m6A-driven genes regulated by METTL3 are functionally relevant. (TIFF) [file pcbi.1005287.s003.tiff]

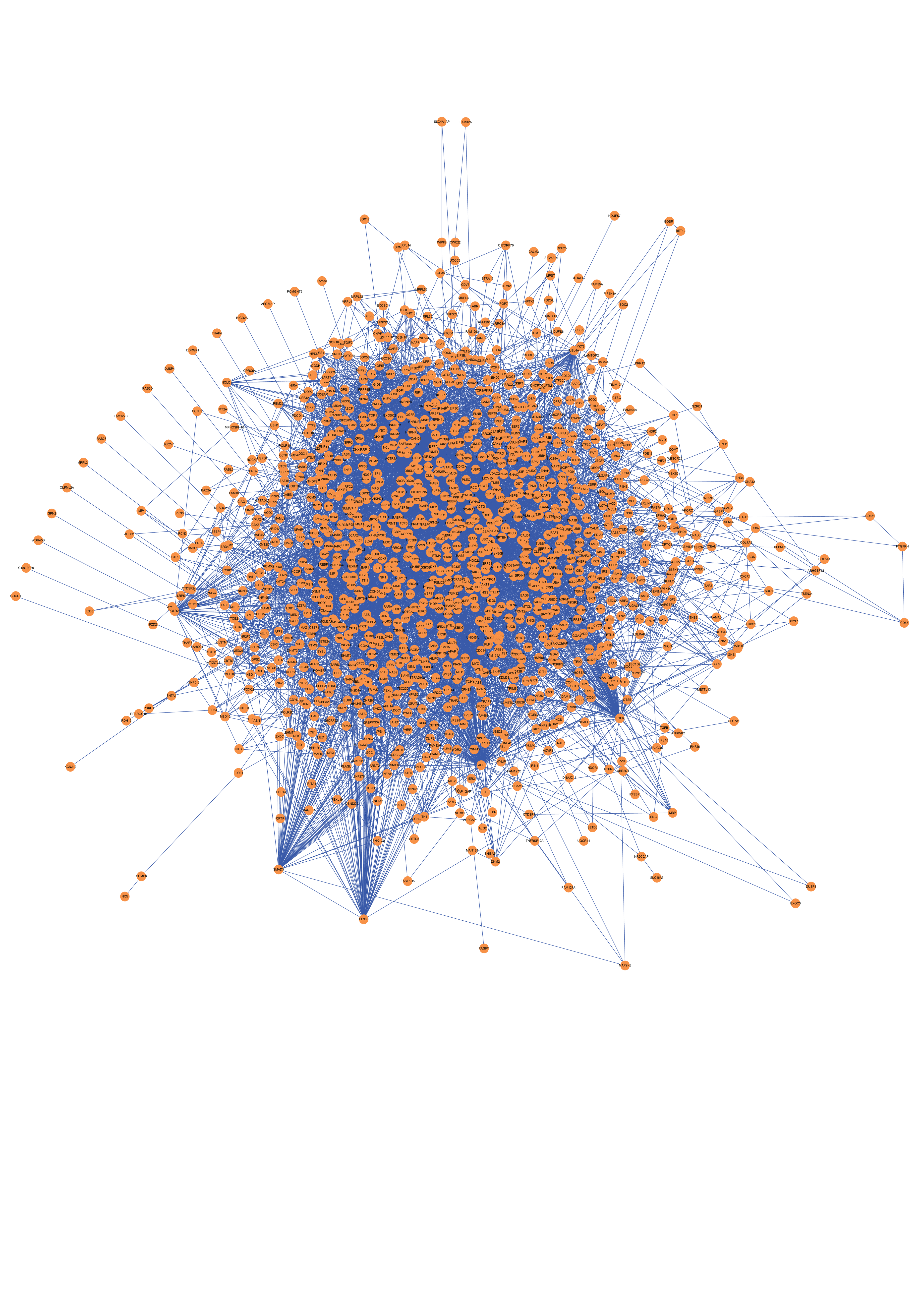

Supplement: S4 Fig — The network consists of m6A-driven genes identified in KD-METTL14 dataset. We can see that they are closely interacted with each other in the network which indicates that m6A-driven genes regulated by METTL14 are functionally relevant. (TIFF) [file pcbi.1005287.s004.tiff]

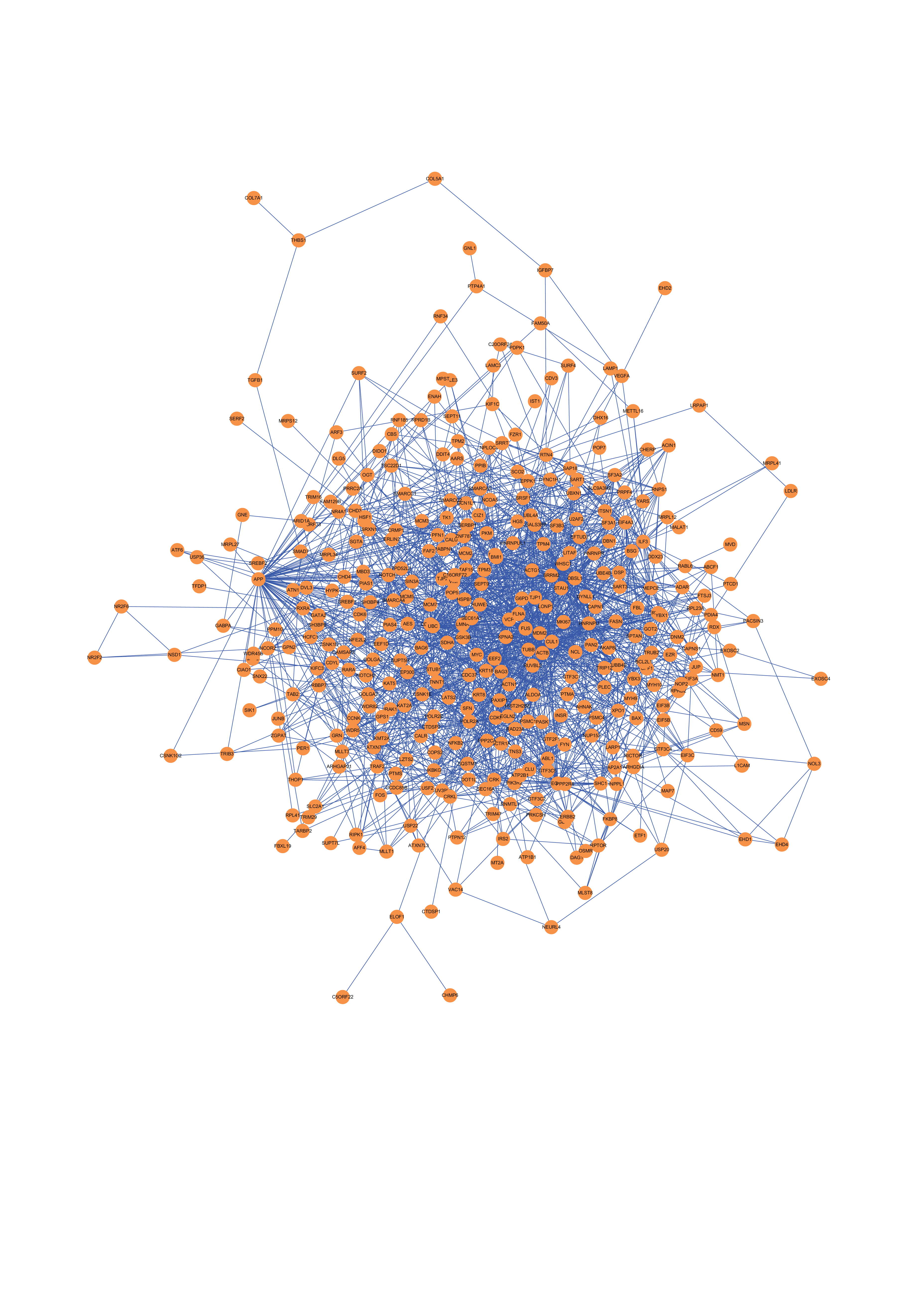

Supplement: S5 Fig — The network consists of m6A-driven genes identified in KD-WTAP dataset. We can see that they are closely interacted with each other in the network which indicates that m6A-driven genes regulated by WTAP are functionally relevant. (TIFF) [file pcbi.1005287.s005.tiff]

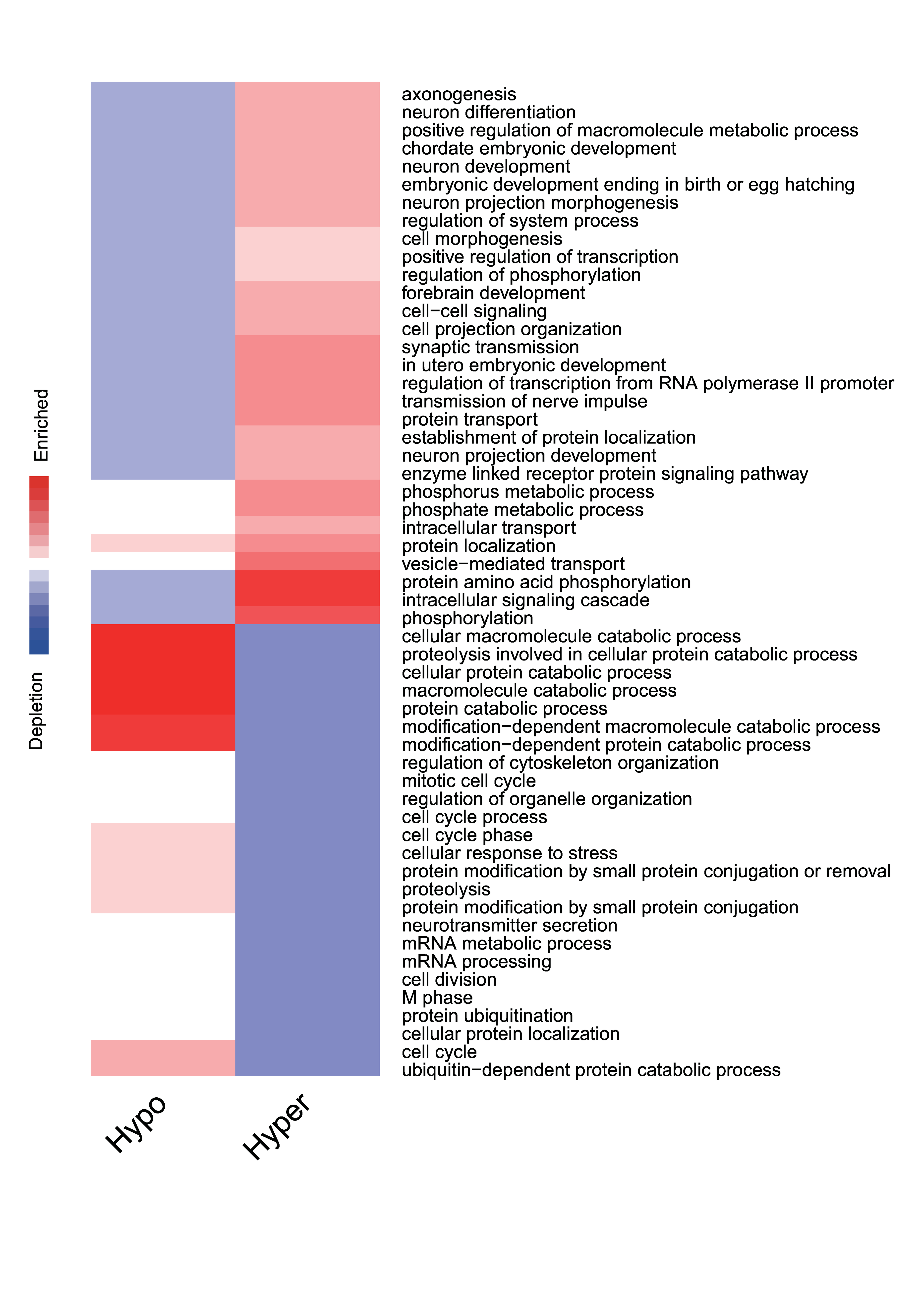

Supplement: S6 Fig — We show here a heat map depicting the GO biological process (BP) categories most enriched in m6A-driven genes identified in KD-FTO dataset. The enrichment analysis is conducted for the hyper and hypo m6A-driven genes respectively using DAVID. The FTO targeted hyper m6A-driven genes closely link to synaptic transmission and cell-cell signaling. And we also find several other significant biological processes and genes regulated by m6A such as embryonic development and neuron differentiation. Thus demonstrates m6A-Driver could identify biological functionally significant m6A-driven genes. (TIFF) [file pcbi.1005287.s006.tiff]

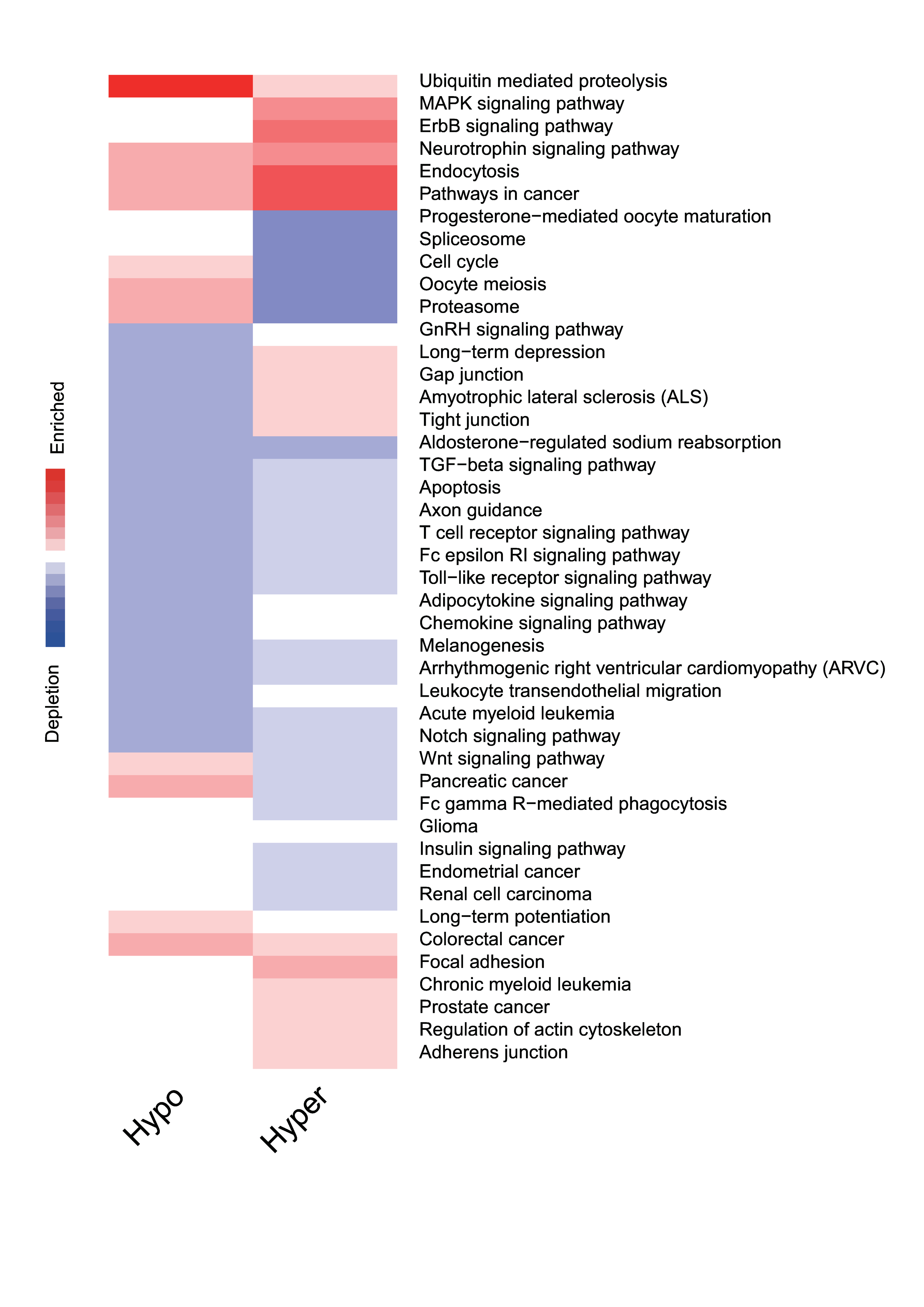

Supplement: S7 Fig — We show here a heat map depicting the KEGG categories most enriched in m6A-driven genes identified in KD-FTO dataset. The enrichment analysis is done to the hyperand hypo m6A-driven genes respectively using DAVID. The m6A-driven genes are significantly enriched in cancer related pathway and some specific cancer such as chronic myeloid leukemia and Glioma which suggest RNA methylation may play a role in cancer. (TIFF) [file pcbi.1005287.s007.tiff]

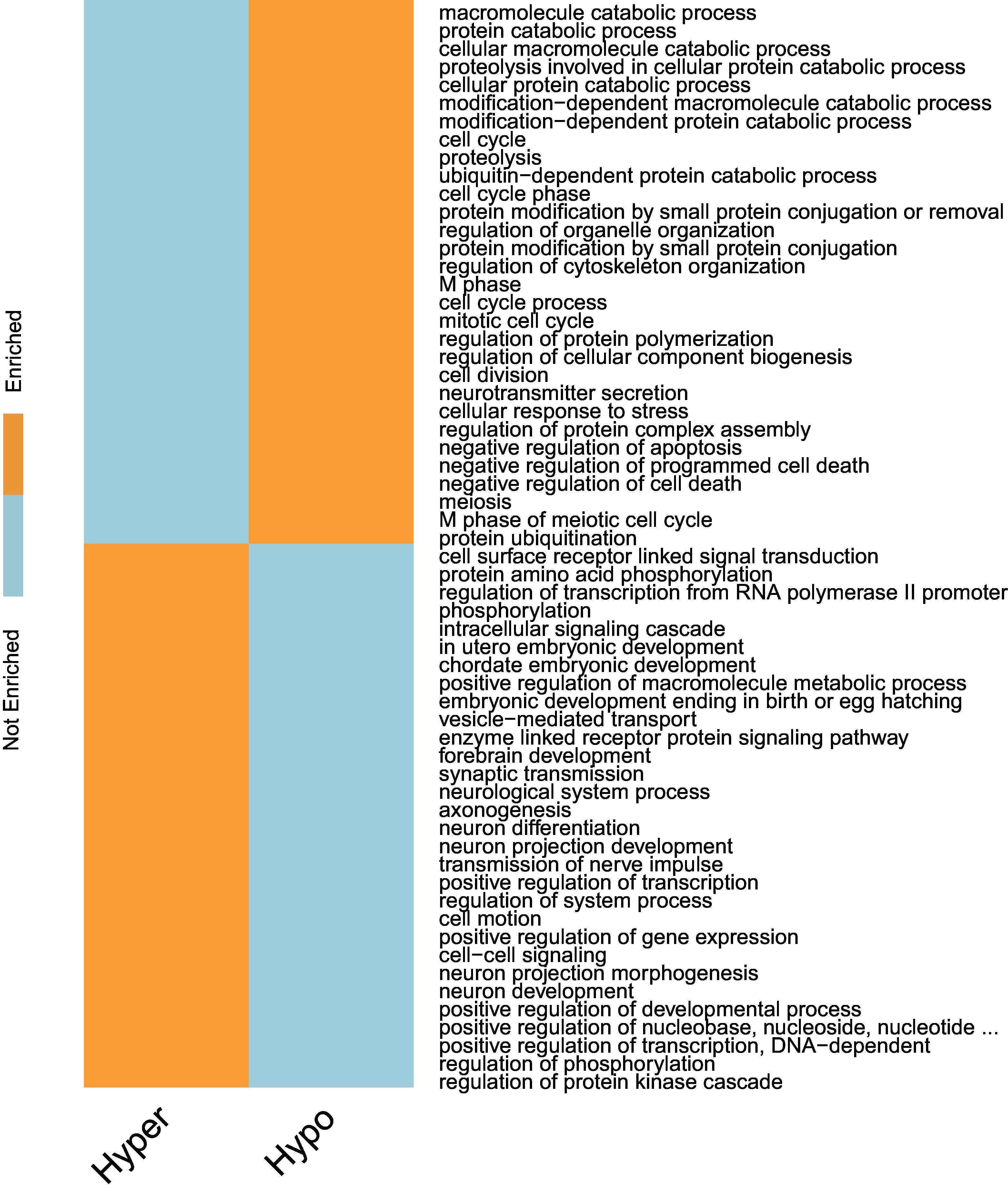

Supplement: S8 Fig — We show here a binary map depicting the GO biological process (BP) categories enriched in m6A-driven genes identified in KD-FTO experiment. The enrichment analysis is conducted for the hyper and hypo m6A-driven genes respectively using DAVID and adopting the brain tissue specific expressed genes as control data.Brain tissue specific expressed genes are genes who have a RPKM value over 1 in at least half of the input samples, including treated and untreated ones. (TIFF) [file pcbi.1005287.s008.tiff]

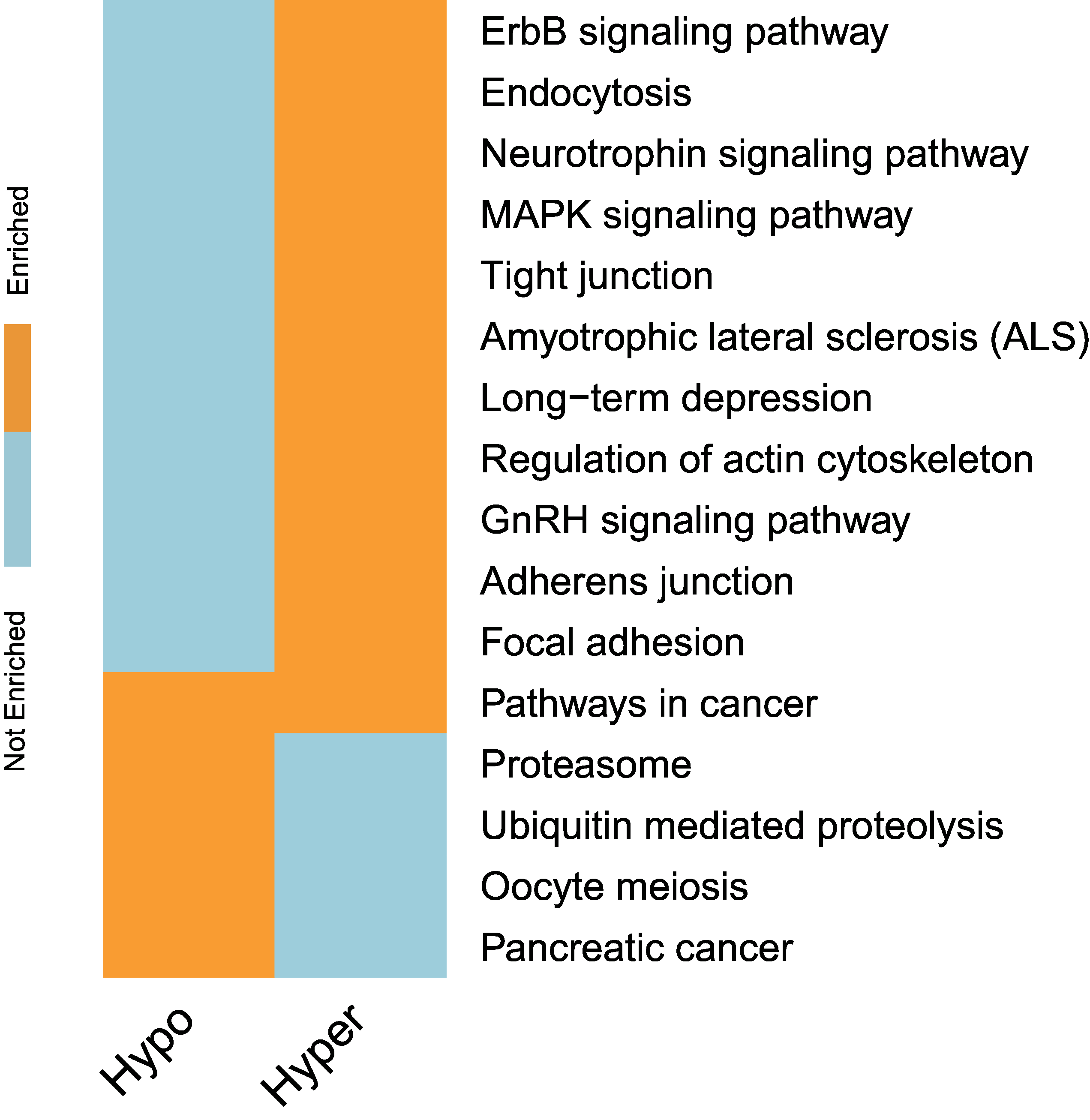

Supplement: S9 Fig — We show here a binary map depicting the KEGG categories most enriched in mDrGenes for KD-FTO dataset. The enrichment analysis is conducted for the hyper and hypo m6A-driven genes respectively using DAVID and adopting the brain tissue specific expressed genes as control data. Brain tissue specific expressed genes are genes who have a RPKM value over 1 in at least half of the input samples, including treated and untreated ones. (TIFF) [file pcbi.1005287.s009.tiff]

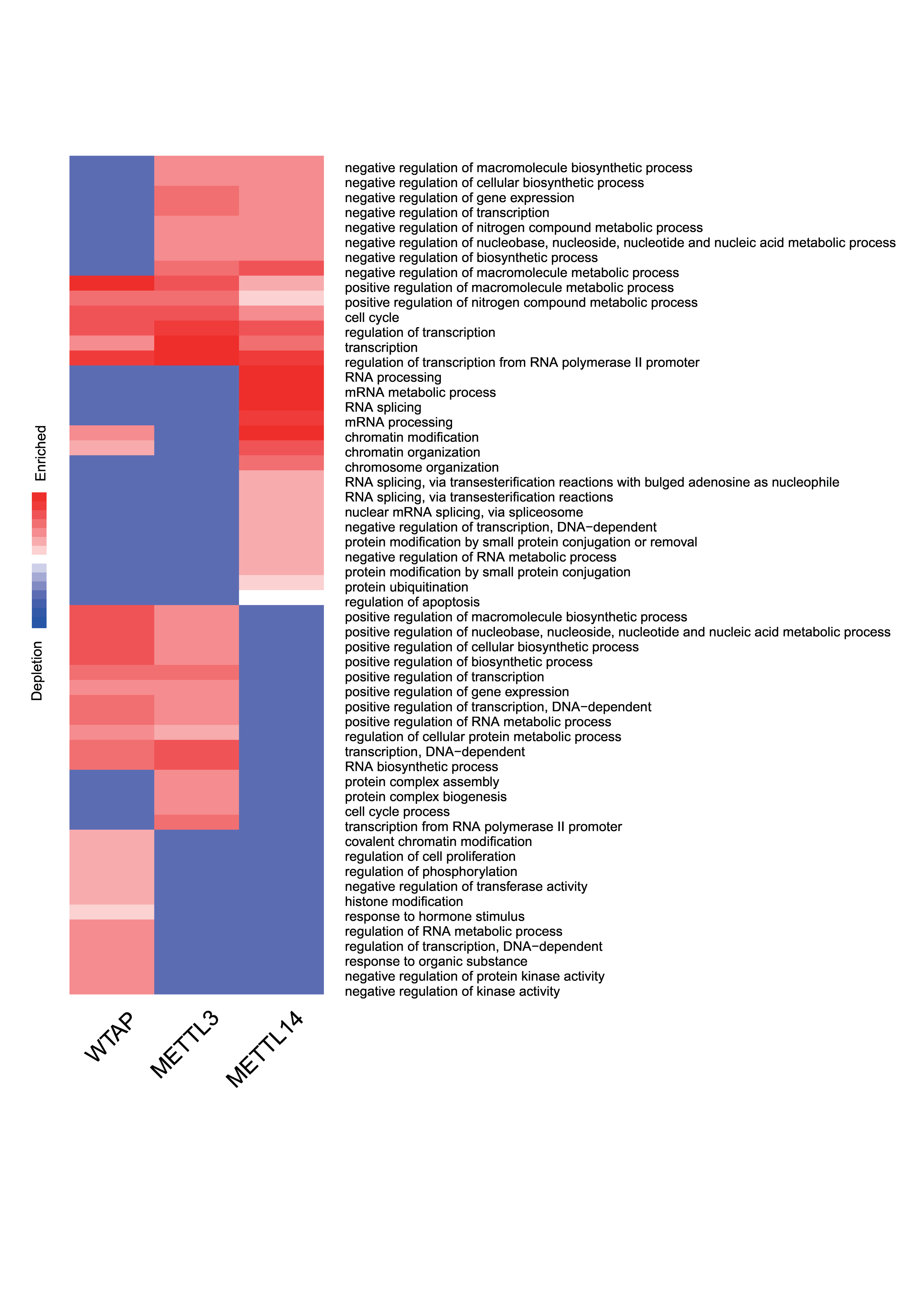

Supplement: S10 Fig — We show here a heat map depicting the GO biological process (BP) categories most enriched in m6A-diven genes identified in KD-METTL3, KD-METTL14 and KD-WTAP using DAVID. There are significant overlapping biological processes between the three enzymes targeted m6A-driven genes and also enzyme specific functions, suggesting that different methylation enzymes may influence different biological processes via driving different genes. (TIFF) [file pcbi.1005287.s010.tiff]

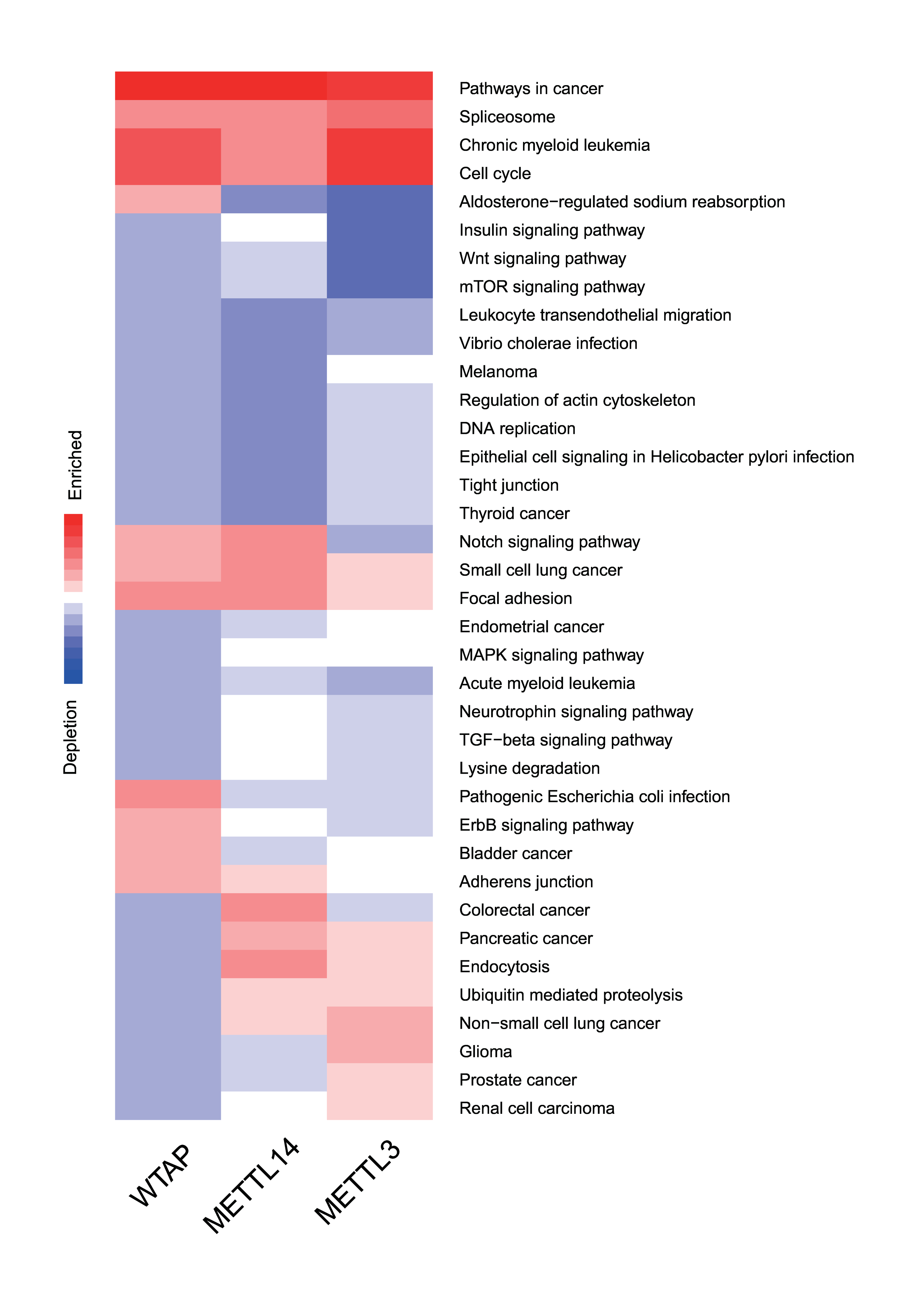

Supplement: S11 Fig — We show here a heat map depicting the KEGG categories most enriched in m6A-driven genes identified in KD-METTL3, KD-METTL14 and KD-WTAP using DAVID. There are significant overlapping pathways between the three enzymes targeted m6A-driven genes and also enzyme specific functions. Two important consistent pathways are cancers and splicing which indicates m6A may regulate these pathways through m6A-driven genes. (TIFF) [file pcbi.1005287.s011.tiff]

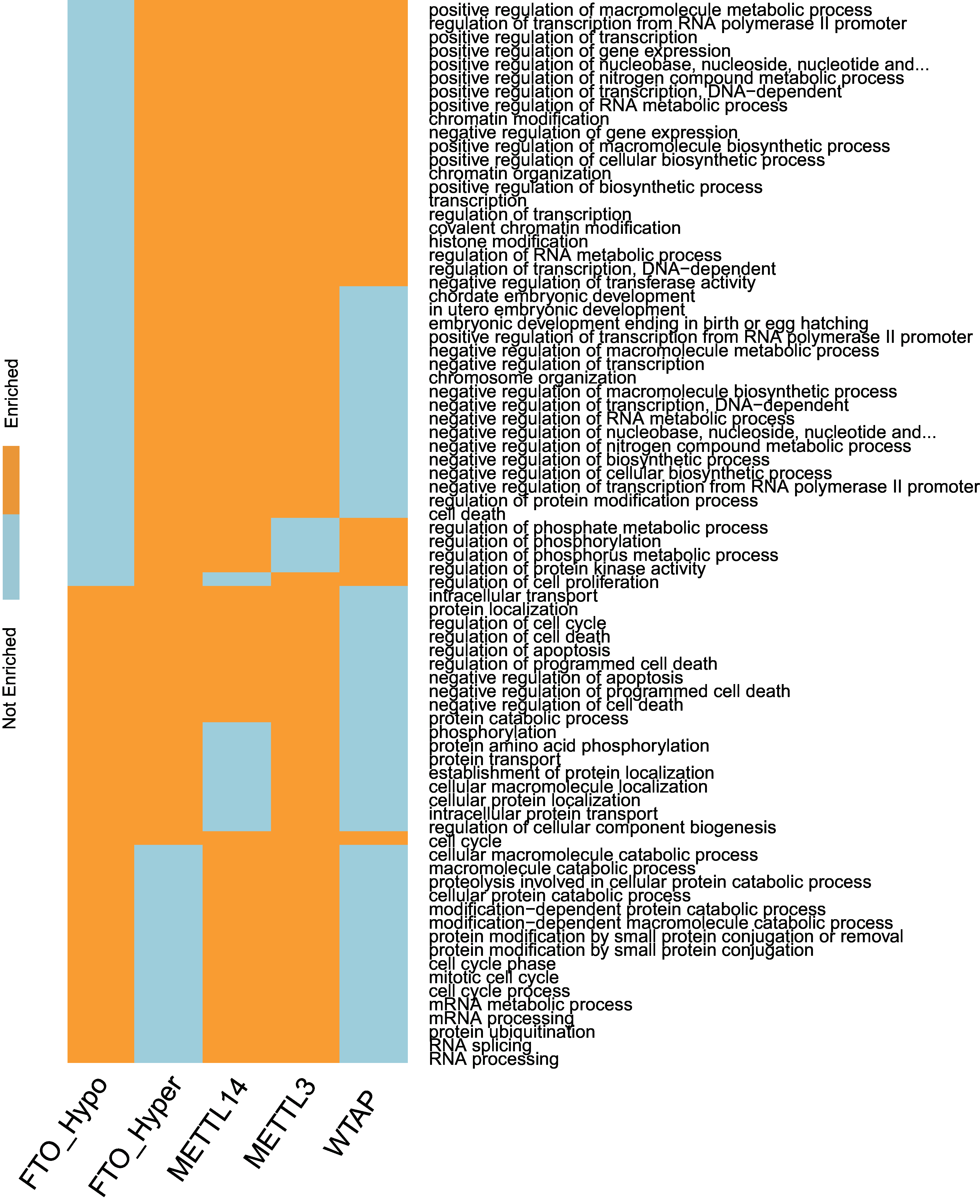

Supplement: S12 Fig — We show here a binary map depicting the GO biological process (BP) categories most enriched in mDrGenes identified in KD-FTO, KD-METTL3, KD-METTL14 and KD-WTAP using DAVID. Enriched BPs in KD-FTO are divided into hyper and hypo groups. We only keep BPs enriched in at least one group (hyper or hypo) of KD-FTO and enriched in at least 3 of the 5 groups to show significant overlap between the 5 group of BPs. The overlap BPs between FTO-hyper group and METTL3/METTL14 are mainly about transcription, regulation of transcription and regulation of gene expression. The overlap BPs between FTO-hypo group and METTL3/METTL14 are mainly about RNA splicing and protein modification. This indicates that the dynamic of m6A may regulate these biological processes in a direct or indirect way. (TIFF) [file pcbi.1005287.s012.tiff]

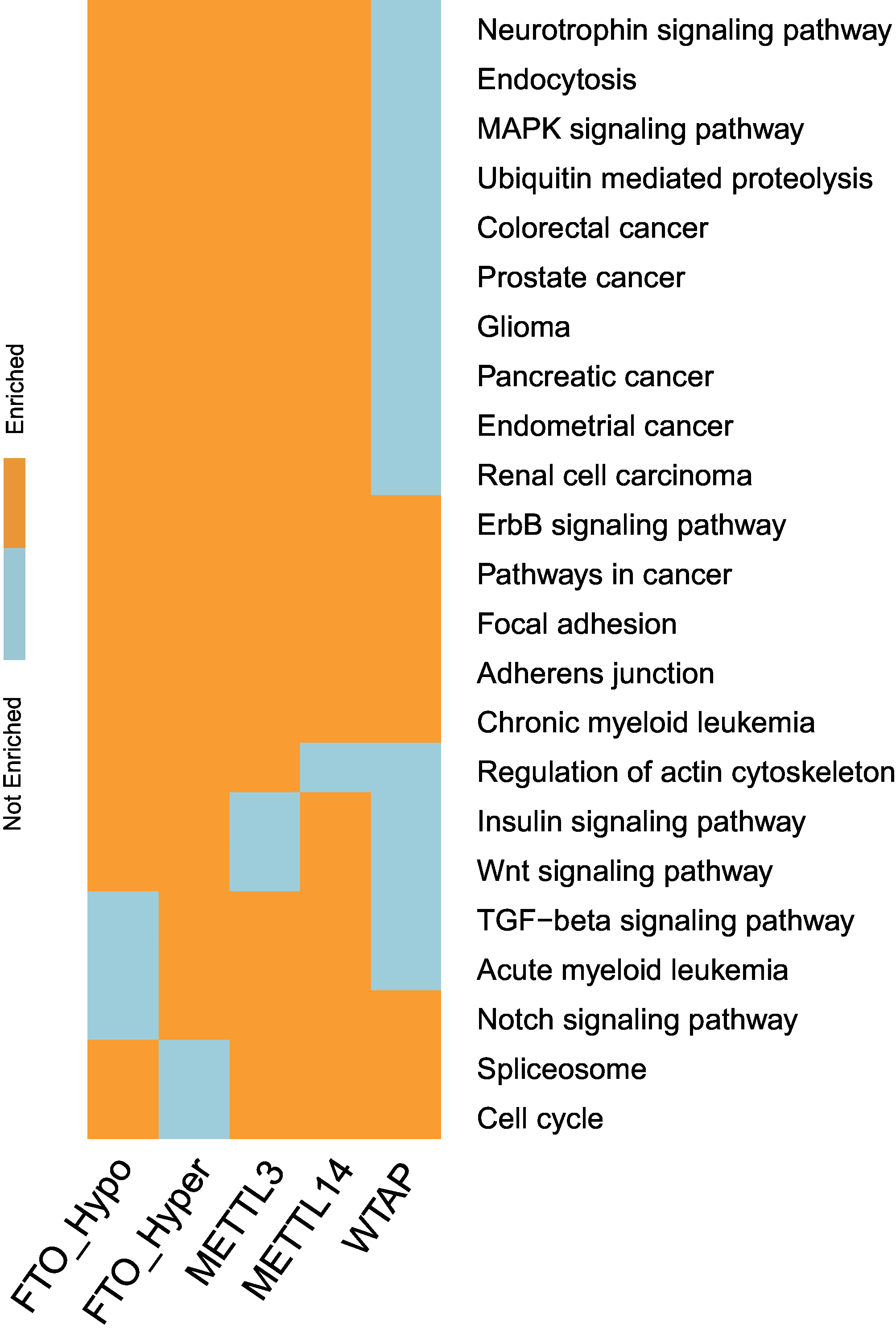

Supplement: S13 Fig — We show here a binary map depicting the KEGG categories most enriched in mDrGenes identified in KD-FTO, KD-METTL3, KD-METTL14 and KD-WTAP using DAVID. Enriched pathways in KD-FTO are divided into hyper and hypo groups. We only keep pathways enriched in at least one group (hyper or hypo) of KD-FTO and enriched in at least 3 of the 5 groups to show significant overlap between the 5 group of pathways. All of the 5 group of mDrGenes are enriched in pathway in cancer, especially in Chronic myeloid leukemia. This further illustrate the dynamic of m6A is related to cancer. The overlap pathways between FTO-hypo group and METTL3/METTL14/WTAP are cell cycle and spliceosome. This is consistent with the result of BP enrichment analysis and confirm the relevance between dynamic m6A and splicing. (TIFF) [file pcbi.1005287.s013.tiff]
